# Microbiome dataset from a marine recirculating aquaculture system (RAS) for salmon post-smolt production in Norway

**DOI:** 10.1016/j.dib.2021.107767

**Published:** 2021-12-26

**Authors:** K. Drønen, I. Roalkvam, H. Dahle, A.B. Olsen, H. Nilsen, H. Wergeland

**Affiliations:** a Department of Biological Science, University of Bergen, Thormøhlensgt 55, N-5020, Norway; b Norwegian Veterinary Institute, Thormøhlensgt 53C, N-5006, Norway

**Keywords:** RAS, Marine post-smolt, 16S rRNA, Microbiomes, Ion Torren

## Abstract

A marine aquaculture recycling system (RAS) for the production of post-smolt was monitored for microbial community structures during the first year of operation. Sample material was obtained monthly from the biofilter biofilm carriers, the production water (tank 3), the fish skin (tank 3) and the tank 3 wall biofilm. Additional samples were taken during outbreaks of fish skin wounds, washing of the plant, UV filtration of the inlet water and from various wall biofilms. Samples for depth profiles from all fish tanks were also collected. The sampling tools were a ladle for capturing biofilter biofilm carriers, toothbrushes for wall biofilm capture, filters for capture of water microbes and scalpels for skin tissue slicing. The sampling times were indicated by the production cycle number (cycle 2-5) and the week number within the cycle (W). Prior to bacterial community analysis, the stored samples were exposed to cell lysis and extraction of environmental DNA by commercial kits. All samples were subjected for PCR amplification of 16S rDNA sequences for library formations and prepared for Ion Torrent technology, which sequences 250 bp fragments. A total of 1.1 million reads were obtained from the 100 RAS samples analysed. The process from Ion Torren analysis to library involved bioinformatics steps with sorting, filtering, adjustment and taxonomic identification, and the final output was shown in a table as operational taxonomic units (OTUs) and relative abundance at different sampling sites and sampling time points. Of a total of 450 taxonomically assigned OTUs, 45% were classified at genus level. The 16S library raw data are deposited in the Mendeley data repository and cited in this Data in Brief article co-submitted with the article “Microbial colonization and stability in a marine post-smolt RAS inoculated with a commercial starter culture.” [1]. So far, the raw data are referenced in four more publications in progress. These cover microbial shifts and enrichments between sampling times, sulfur cycling, “in vivo biofilm” and identification of relatives of fish pathogens in RAS. All library sequences are available in GenBank with accession numbers MN890148-MN891672.

## Specifications Table

**Table:** 

Subject	Biological science
Specific subject area	Microbiology: Microbiome
Type of data	This Data in Brief article includes several figures that presents results from Next Generation Sequencing downstream analyses. The raw data were deposited in GenBank as sequences in a fasta file and the Mendeley data repository as a CSV file. Links to this information are included in the present article. For manual data interpretation, an additional pdf file of the raw data was added the Mendeley data repository along with the CSV file. Here data were sorted by descending relative abundance for each sampling time and sampling site. The relative abundances were visualized by colours for different categories of relative abundance.
How data were acquired	Library data were acquired by molecular methods, starting with environmental DNA as template for PCR amplification of variable 16S regions. Individual sequences in the product were sequenced by Ion Torrent Personal Genome Machine (PGM) technology, and bioinformatics tools sorted and filtered data into OTUs, from where final taxonomic identification was performed using the Qiime platform.
Data format	Amplicon libraries “raw” data are provided as an excel CSV file, and sorted data by relative abundance vs sample are provided as an ordinary excel file termed Appendix. Analysed data are provided as figures in pdf formats.
Parameters for data collection	The data were collected from the first marine RAS for post-smolt, and RAS events were coming forth between the regular sampling times. Major RAS events were skin ulcers formation, antibiotics administration, farm cleaning, back flushing of the biofilter biofilm, re-inoculation of the biofilter, necromass formation, yellow substance accumulation and sharp shifts in pH and salinity.
Description of data collection	Biofilm carriers were collected by a ladle led down into the biofilter chamber. Wall biofilm was collected by toothbrush heads led along the wall of the production tank in study. Wall biofilm was at one sampling time also retrieved from biofilter outer-chamber, the pump sump, and depth profiles from all production tanks. Water samples were retrieved using 0.22um Sterivex filters.
Data source location	Institution: University of Bergen City: Bergen Country: Norway Sample location: Erko settefisk AS, Stord, Norway (59°46′20.61″N 5°23′0.98″E)
Data accessibility	Repository name: Mendeley Data Data identification number: doi: 10.17632/wff3kn8zxc.4 Direct URL to data: http://dx.doi.org/10.17632/wff3kn8zxc.4 All library sequences are available at GenBank with the accession numbers MN890148-MN891672.
Related research article	Co-submitted paper: K. Drønen, I. Roalkvam, H. Dahle, H. Nilsen, A.B. Olsen, H. Wergeland, Microbial colonization and community stability in a marine post-smolt RAS inoculated with a commercial starter culture, Aquac. Rep. 20 (2021) 100745. https://doi.org/10.1016/j.aqrep.2021.100745.

## Value of the Data

• This study contributes to the holistic thinking around the microbiology of marine post-smolt RAS technology. The data address questions about the dynamics and degree of variation in the RAS microbiomes that can be related to physiological and chemical water parameters and operational events otherwise reported. The combined information can be used for understanding how to achieve long-term good water quality and fish health in RAS.
• The data are requested by the Norwegian fish farming industry and by the Norwegian fish health authorities.
• The descriptive data can be interpreted in the context of theoretical ecology and from here form hypotheses to be tested by empirical studies.
• For enhanced holistically understanding of the RAS microbiome, suggested major processes should be verified by transcriptomics.

## 1 Data Description

**Fig. 1:**
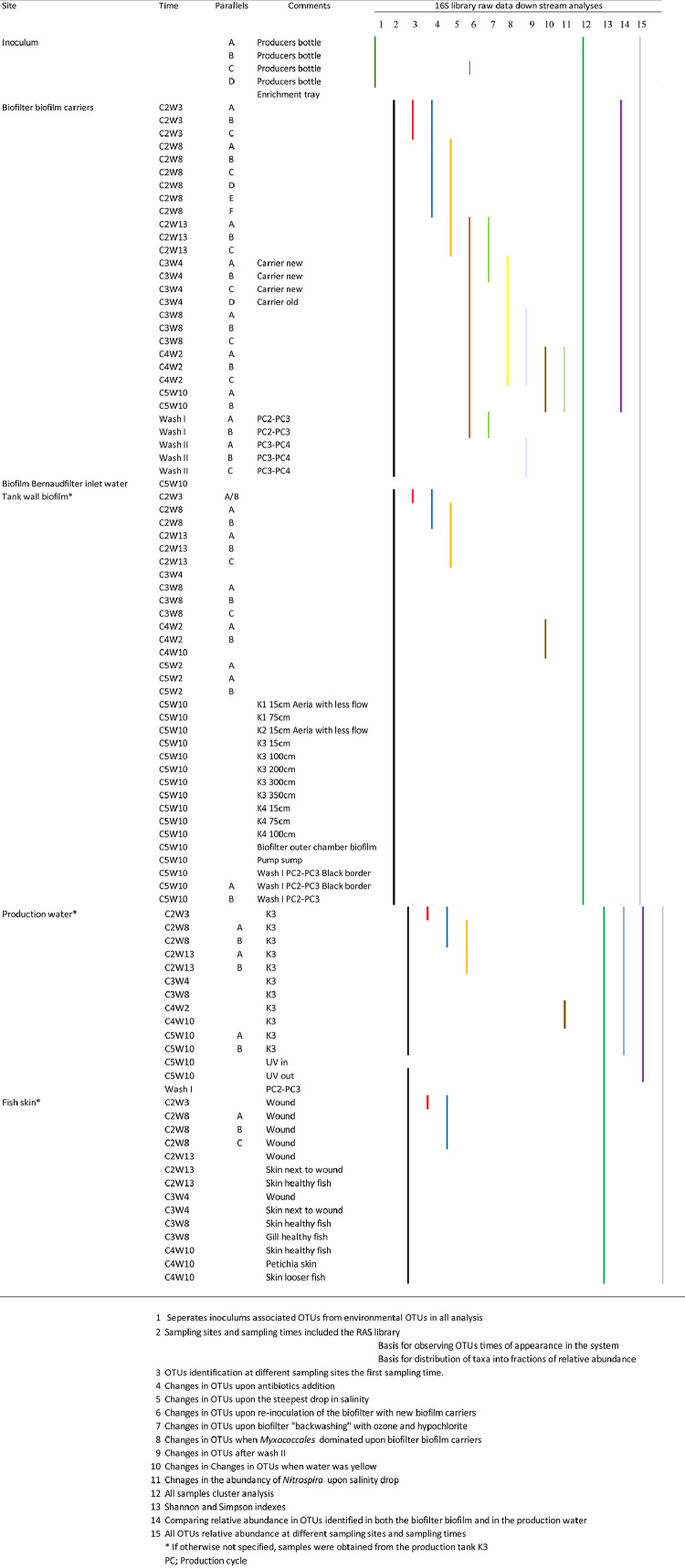
Overview of the 16S rRNA library from a marine post-smolt RAS; sampling sites, sampling times and usage in analyses (coloured lines).

The amplicon analysis´s raw data are deposited to the Mendeley database and represent 90 environmental samples from a marine RAS [2]. The samples originated mainly from biofilter biofilm carriers, tank wall biofilm, production water and fish skin. Parallel samples were collected for the biofilms. An overview of the library´s sampling material and usage in analysis is shown in Fig. 1. The sampling times were denoted by cycle number (C2-5) and week number within the cycle (W).

In addition to samples from regular sampling sites, five replicates from the commercial starter culture used for inoculation of the biofilter biofilm carriers were also analysed. These data made the basis for separation between inoculum associated OTUs (IAO) and other (‘environmental’) OTUs. Biofilm samples were also collected at the final sampling time point (C5W10), from biofilter outer chamber wall, the pump sump wall and from all production tank walls at various depths. The water in and out of the UV-filter was also sampled at this point (Fig. 1, see comments lower part of wall samples).

**Fig. 2:**
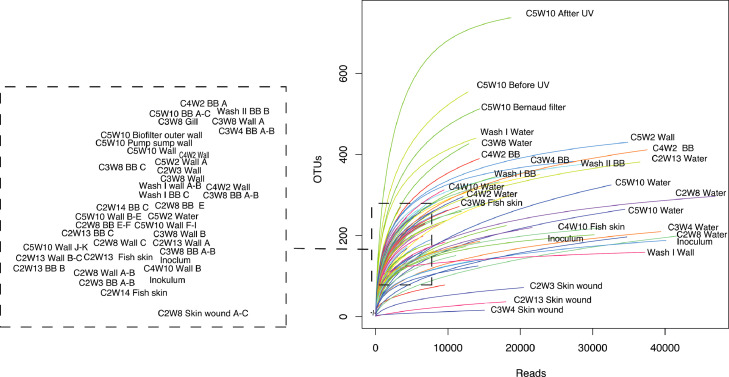
Rarefaction curve. BB; Biofilter biofilm carriers, Water; Production water, Wall; Tank wall biofilm. Rarefaction curves are created by randomly re-sampling the pool of N samples multiple times and then plotting the average number of species found in each sample at a given sampling depth (indicated on the x-axis).

“Secondary Data”:

The amplicon data´s rarefaction curves are given in Fig. 2, and are used for evaluating of methodical approaches with respect to depth of sequencing.

**Fig. 3:**
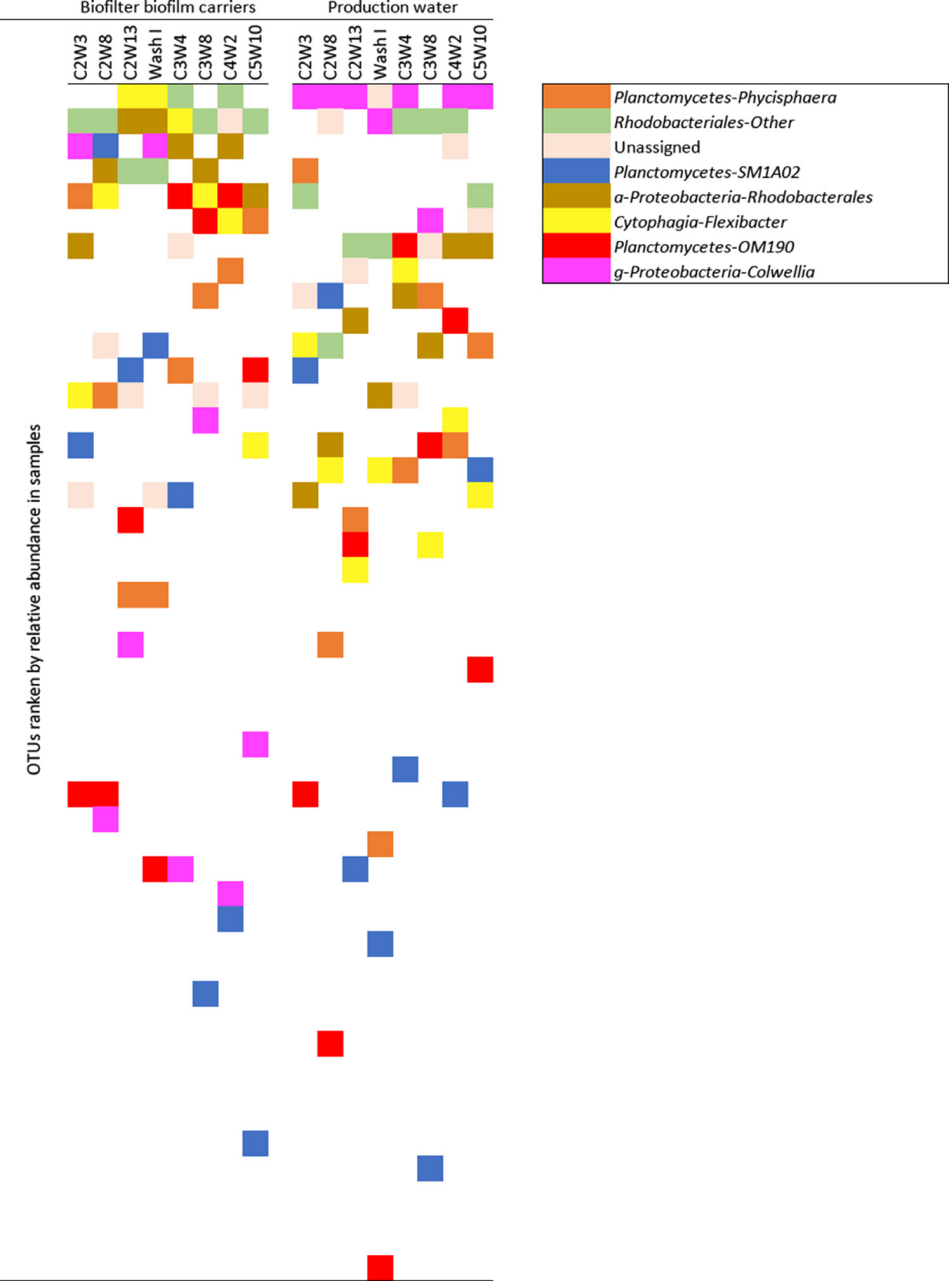
Pattern recognition of the dominat bacterial genera in the biofilter biofilm back in the production water. The pattern is shown for the eight mutual sampling times between the two sampling sites. The ranking was given by their placement in the descending relative abundance line.

**Fig. 4:**
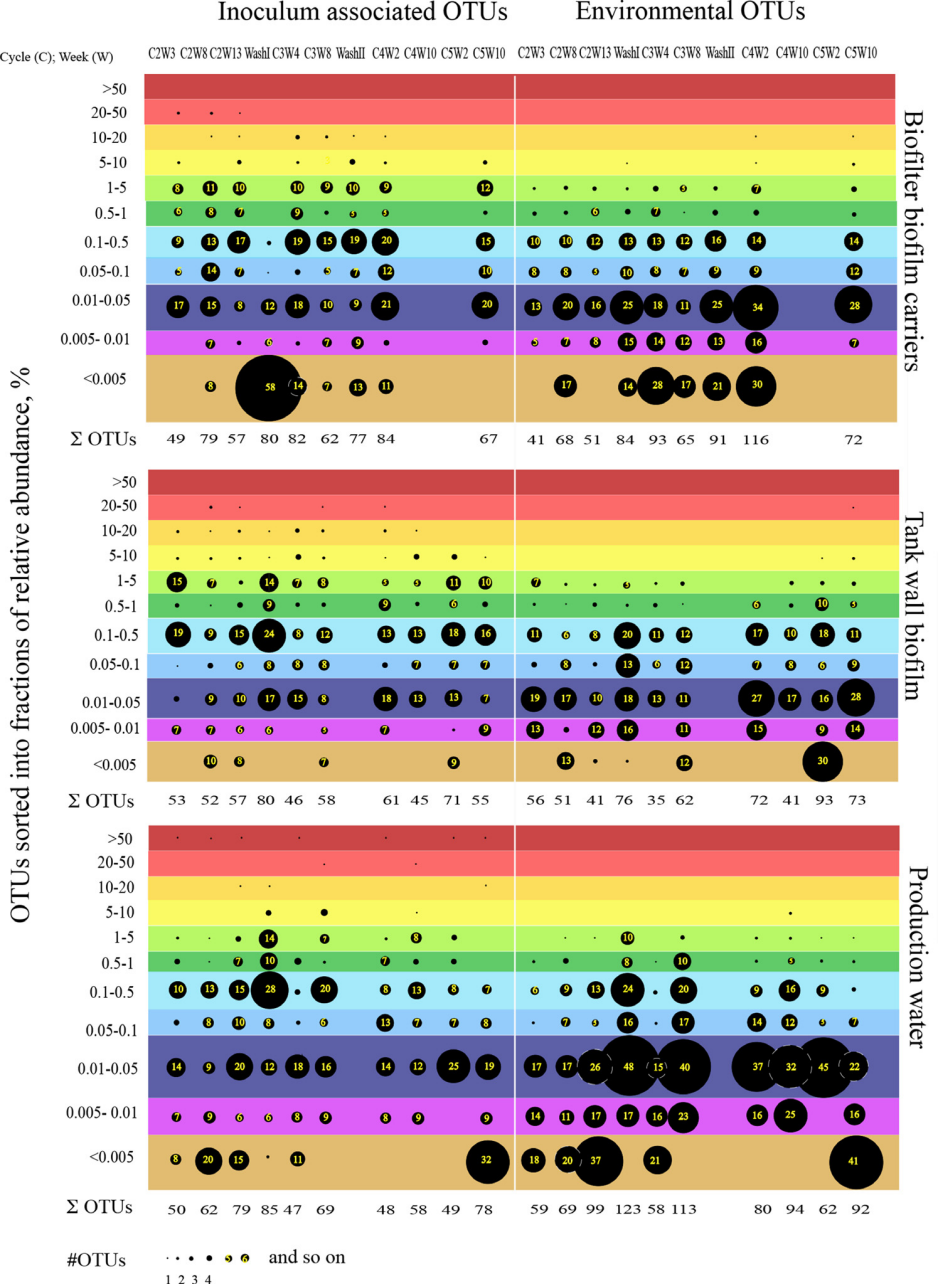
Distribution of taxonomically identified RAS OTUs in 11 categories of relative abundance at the different sampling sites and sampling times. Data are shown for biofilter biofilm carriers, the tank wall biofilm and the production water. The OTU data are sorted by origin, either starter culture or environment.

The deposited appendix file also presents amplicon libraries with raw sequencing data sorted by sampling sites and relative abundance [2]. This was used to identify dominant OTUs, but also to perform relative abundance pattern recognition of shaded biofilter biofilm in the production water (Fig. 3). These OTU data were also extracted and presented in the bubble plot in Fig. 4. This figure show the OTU data for each sampling site and sampling time, based on pooled replicate samples data and unrarefied data.

**Fig. 5:**
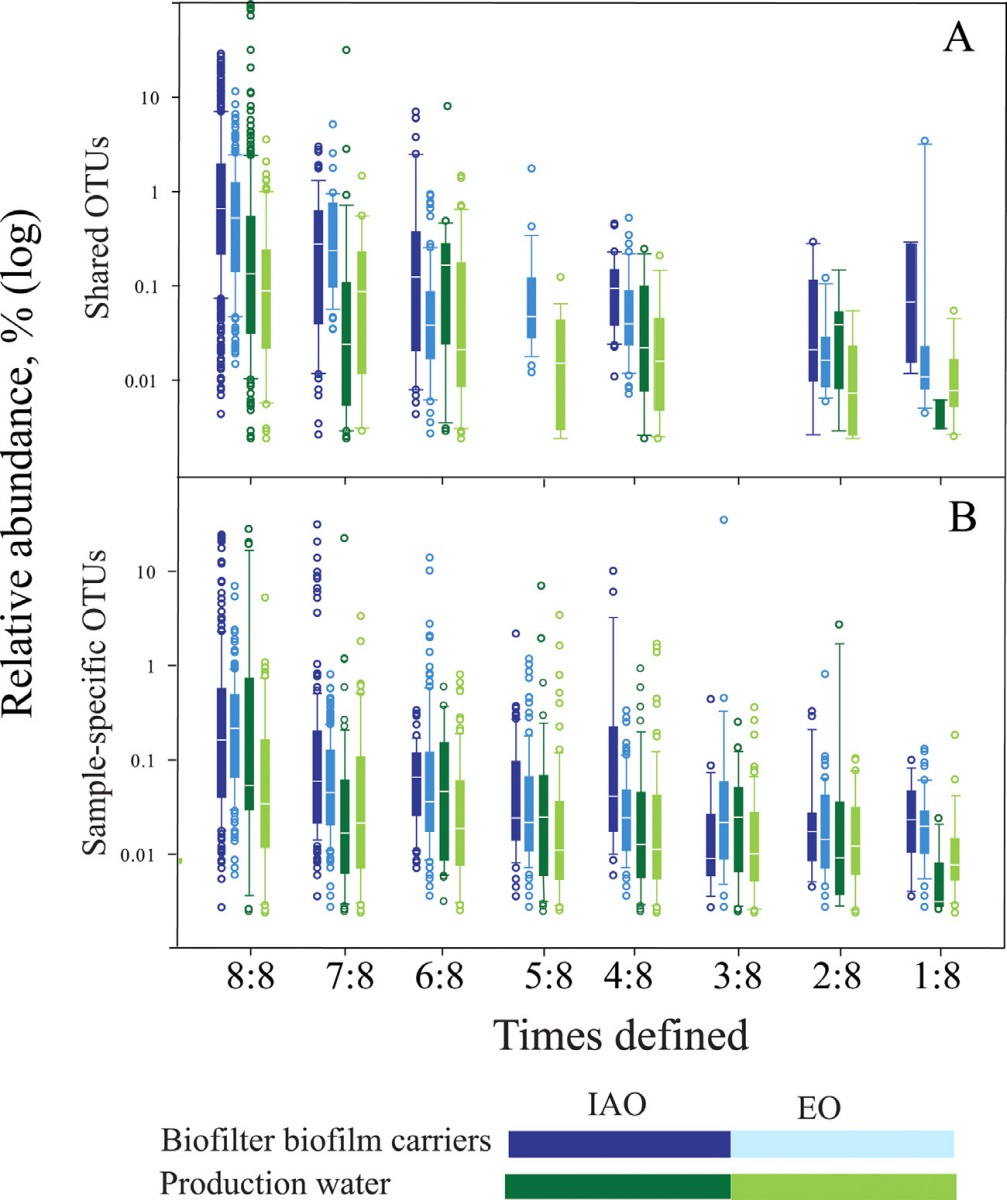
Boxplot of relative abundance of shared (A) and sample-spesific (B) OTUs from biofilter biofilm carriers and production water versus times of appearance. IAO; inoculums associated OTUs, EO; environmental OTUs. The median is shown as white lines between the 25- and 75-percentiles. The logarithmic scale of the relative abundance (%) shows the 25-percentile relative smaller than the 75-percentile, as compared to a linear plot. The whiskers show minimum and maximum values, i.e., the lowest and highest datapoints that exclude outlier. Open dots are outliers. Data are based on OTU data from all samples and data are not rarefied [2].

## 2 Experimental Design, Materials and Methods

### 2.1 Samples retrieved from the RAS

Water microbes were sampled into a Milipore Sterivex^TM^ filtre GV 0.22mm by injecting 240ml water with a syringe (60ml, BD Plastipak^TM^) through the filter. The surrounding plastic caging was filled with RNAlater to enhance RNA and DNA stability of the biological material during storage (http://patft.uspto.gov patent #6,528,641, 2mM EDTA, 25mM sodium citrate, 5.3mM ammonium sulphate, pH5.2). Wall biofilm (3 parallels) was collected by toothbrushes (new from box) that were led horizontally along the tank wall (10meters) 15cm below the water surface on a long rod. The brush head was stored in 15ml RNAlater in a 50ml Falcon tube. Biofilter biofilm carriers (3 parallels) were collected (ladle) and transferred (tweezer) sterile into a 50ml Falcon tube with 10 ml RNAlater. Prior to fish skin sampling, the fish was killed by a sharp blow to the head. Fish skin samples, 2 × 3cm and 1mm thick, were cut out with a scalpel in the area laterally to the dorsal fin and transferred with a sterile tweezer into a 50ml Falcon tube with 10ml RNAlater. Skin samples were not paralleled, except at sampling time C2W8, when three samples were taken from the same skin wound. The samples were kept cold during transportation and toothbrushes and biofilm carriers were sonicated (3 × 30sec) in water bath to assist the biofilm loosening. The brush heads and carriers were removed and the organic material pelleted by centrifugation for 45min 5000g 7 °C (Heraeus, Scientific Centrifuge (3SR+)) prior to storage at −24 °C.

### 2.2 DNA extraction

DNA was extracted from the samples using kits designed for the different sample types. All the kits processed lysed material by protein removal, DNA capture in columns, washing and elution, but differed in the initial disruption of the cells. DNeasy PowerBiofilm Kit (Qiagen) was used for biofilter biofilm samples and combined enzymatic cell lysis with mechanical disruption (bead beating). DNAeasy PowerWater kit (Qiagen) supported beat beading tubes that were designed in particular for the Sterivex filters. These were loosened from the plastic caging by tongs and sterile scalpel. The tissue DNA extraction kit delivered by Rocke (High Pure PCR Template Preparation) was used for the fish skin samples. In this kit, the lysis was ensured by long time-high temperature exposure of 0.2g tissue.

### 2.3 Microbial deep sequencing and 16S amplicon library

The 16S amplicon rRNA libraries were generated in accordance with the protocol of Jørgensen et al. 2016 [4] and modified after the two-step amplification protocol recommended by Berry et al. 2011 [3]. These protocols prepared for subsequent library sequencing in the Ion Torrent Personal Genome Machine (PGM) platform technology [5]. For the first-step rDNA amplification we used the primers 519f (5’-CAGCMGCCGCGGTAA-) and 805r (5’-GACTACHVGGGTATCTAATCC) in the PCR mixture containing 12.5µl HotStarTaq Master Mix Kit (Qiagen), 2µl of each primer (100mM), 7.6µl dH_2_O and 2µl DNA-template. The thermal cycle program was: 95 °C, 15min, 32 times repeating DNA melting (94 °C, 30s); primer annealing (56 °C, 30s) and amplification (72 °C, 30s). Positive PCR products were verified by 1% agarose gel electrophoresis (Agarose Electran, Cambrex Bio Science, 50V, 30min), using 1µl GelGreen Nucleic Acid Stain per 10ml agarose (Biotium, VWR). DNA templates with positive product were triplicated in parallel PCR amplifications, before pooled triplicate DNA samples were purified by the bead based AMPure XP kit (Agencourt) using a 96 well square storage plate 1.2ml (Thermo scientific, AB-1127) adaptable to a magnet plate (Alphaqua, 96S Super). We held the sample:bead ratio (0.7), ethanol washing solution (70%) and H_2_O eluation volume (20µl) in accordance with supplier's manual. The purified DNA (2ul) was quantified in a fluorometer (Quantus, Promega) using a fluorochrome (0.5µl, QuantiFluor® dsDNA System mixture) and 1xTE buffer (197.5µl). The kit provided also a DNA standard (100ng/µl). Samples were diluted to a final concentration of 10ng/µl as template for a second PCR with tagged primers: Forward primer was tagged with a start site for the DNA polymerase and a barcode flag (Multiplex Identifiers, MIDs) and a code for PGM calibration [5]. Reverse primer was tagged with the “adapter B sequence” for bead attachment in the PGM operation. The PCR mix of the second PCR was as following: 2.5µl HotStarTaq Master Mix Kit, 0.3µl H_2_O, 2µl 519f MID primer (10µM) and 0.2µl 806r B-key primer (100µM) and 10µl template (10ng/µl). Differed from the initial PCR, the number of cycles were 7, but primers and primer-dimers formation were still checked in agarose gel. Purified and quantified product was then ready for two step normalization: First 8 and 8 samples were pooled to their mean, and then the 12 samples were pooled to a concentration of 0.1ng/µl. This stock was quantified prior to the final dilution (40pM) the very day of sequencing.

### 2.4 Sequencing, bioinformatics and data handling

Amplicons were sequenced on a PGM in the Laboratory of Bioscience, University of Bergen, Norway. The down-stream 16S rRNA gene sequence analysis includes the following steps: Filtering and clustering of sequences into operational taxonomic units (OTUs) using USEARCH and UPARSE. [6,7] Quality filtering and trimming to 250bp with the ‘-fastq filter’ command using the options ‘-fastq_trunclen 250’ and ‘-fastq maxee 1’. Chimeric sequences were detected and removed with the ‘-uchime_ref’ command using the Gold database as reference (available from ‘http://drive5.com/uchime/gold’). De novo OTU clustering was performed at a cut off of 97% nucleotide sequence similarity using the‘-cluster_otus’ command. Taxonomic classification was performed in QIIME, using the command ‘summarize_taxa_through_plots.py’ using Silva 128 as reference database [8]. An overview of the 90 samples represented in the RAS library is given in Fig. 1, and the library sequences are available in GenBank with the accession numbers MN890148-MN891672. The amplicon data refraction curve was made in R by the ‘vegan’ package [9], whereas the appendix file was made in Excel, both based on the raw sequencing data. Figures otherwise were made in excel, sigmaplot and Illustrator.

## Ethics Statement

The study used fish collected from ordinary production cycles at the fish farm, which are not under the act of animal ethic legislation concerning use of animals in Norway. Therefore, no ethical committee is required. Investigated fish were killed according to the Norwegian law, as described above.

## CRediT Author Statement

**K. Drønen:** Did sampling at the location, performed DNA extractions and amplicon library preparations, did the ecological analyses and interpretation of data, and wrote the manuscript; **I. Roalkvam:** Did sample at the location, was involved in the interpretation of the data, and revised the manuscript; **H. Dahle:** Provided bioinformatics tools for sorting, cleaning, and taxonomical classification of the amplicon reads, and also helped in the analysis and interpretation of the data, and revised the manuscript; **H. Nilsen, A.B. Olsen** and, **H. Wergeland:** Revised the manuscript.

## Declaration of Competing Interest

The authors declare that they have no known competing financial interests or personal relationships which have or could be perceived to have influenced the work reported in this article.

## Appendix Supplementary materials

Image, application 1

Image, application 2
