# Supplementary material for: Microbiome dataset from a marine recirculating aquaculture system (RAS) for salmon post-smolt production in Norway
Source: Data Brief. 2021 Dec 26;40:107767. doi: 10.1016/j.dib.2021.107767 (PMC8718742; doi:10.1016/j.dib.2021.107767)
Supplement: Supplementary file 2 [file mmc2.pdf]

APPENDIX

1-10 Relative abundance of taxa sorted largest to smallest

11-12 Taxa number codes.

APPENDIX -1: Inoculums associated taxa in biofilter biofilm.

C2W3		C2W8		C2W13		Washl		C3W4		C3W8		C4W2		C5W10	
11	22,784	11	21,068	86	24,075	86	14,294	71	13,398	134	18,150	71	18,786	106	7,907
71	20,072	71	20,898	34	13,454	34	13,071	86	13,267	71	15,547	12	7,047	71	6,707
95	9,837	80	12,228	56	8,552	95	11,229	34	12,709	65	9,120	34	5,971	134	5,810
104	7,989	34	4,806	71	7,875	71	6,792	134	8,131	34	7,697	108	4,562	65	4,045
13	4,762	86	3,930	11	6,757	56	3,821	30	6,535	86	5,803	30	4,355	34	3,512
94	3,336	22	2,615	120	2,215	61	3,762	11	2,526	30	4,976	86	4,069	13	3,290
34	3,202	133	2,441	22	1,952	133	3,491	12	2,318	108	3,733	18	2,047	42	3,040
22	3,006	38	2,346	133	1,865	120	2,648	18	2,029	126	3,562	13	1,337	104	3,017
18	2,876	1	2,269	18	1,830	57	2,232	108	2,013	13	3,199	57	1,263	22	2,668
17	2,621	57	1,905	66	1,652	17	2,150	96	1,782	104	2,795	61	1,165	108	2,490
96	2,176	12	1,675	124	1,605	80	2,006	85	1,717	70	1,774	11	1,152	96	2,390
99	1,295	120	1,411	80	1,494	124	1,848	13	1,639	96	1,646	96	1,018	30	2,142
86	0,996	13	1,411	12	1,349	99	1,548	65	1,410	12	1,543	106	0,974	12	2,112
56	0,872	99	1,013	61	1,021	11	1,477	66	1,343	95	1,097	54	0,786	66	1,660
80	0,688	66	0,992	126	1,006	66	1,135	54	1,251	17	0,736	133	0,715	86	1,124
124	0,583	18	0,908	57	0,959	131	1,057	61	0,917	66	0,721	38	0,654	18	0,978
12	0,561	124	0,745	99	0,733	12	0,689	80	0,909	85	0,619	16	0,634	85	0,958
70	0,548	17	0,633	30	0,714	54	0,684	38	0,841	56	0,322	128	0,490	56	0,584
115	0,333	104	0,603	128	0,648	128	0,620	17	0,835	11	0,293	22	0,475	57	0,496
133	0,284	96	0,566	55	0,601	25	0,576	56	0,757	128	0,284	134	0,453	128	0,385
119	0,226	61	0,564	13	0,546	13	0,572	57	0,655	18	0,273	116	0,450	54	0,383
66	0,177	94	0,502	134	0,501	18	0,557	133	0,653	54	0,266	66	0,407	38	0,377
116	0,174	111	0,429	95	0,480	85	0,471	55	0,595	55	0,235	99	0,389	139	0,357
111	0,158	2	0,360	96	0,465	138	0,443	128	0,500	110	0,212	17	0,330	126	0,349
108	0,137	32	0,222	54	0,363	83	0,411	22	0,474	99	0,202	25	0,305	124	0,343
76	0,132	85	0,216	94	0,362	94	0,371	70	0,456	22	0,197	124	0,296	61	0,343
61	0,125	25	0,214	104	0,347	55	0,341	116	0,453	57	0,180	72	0,273	95	0,337
9	0,099	108	0,205	25	0,298	111	0,338	104	0,424	38	0,172	85	0,269	116	0,321
30	0,090	30	0,194	38	0,237	91	0,337	111	0,337	61	0,143	41	0,252	72	0,228
57	0,087	95	0,179	131	0,235	96	0,318	41	0,331	111	0,117	1	0,235	133	0,206
137	0,081	54	0,172	17	0,234	22	0,294	25	0,321	115	0,117	64	0,231	110	0,182
120	0,080	119	0,161	110	0,229	30	0,294	95	0,310	133	0,112	111	0,220	120	0,173
85	0,035	116	0,148	111	0,224	16	0,215	120	0,306	124	0,097	95	0,220	48	0,099
83	0,035	70	0,145	70	0,175	9	0,210	99	0,280	41	0,088	80	0,199	11	0,095
67	0,035	9	0,119	85	0,164	130	0,199	48	0,271	120	0,084	104	0,148	68	0,085
27	0,032	131	0,096	116	0,139	119	0,187	115	0,256	16	0,082	120	0,117	99	0,079
65	0,026	76	0,096	42	0,139	29	0,187	16	0,254	80	0,060	56	0,103	27	0,073
129	0,026	6	0,091	29	0,115	115	0,175	124	0,252	106	0,045	136	0,099	109	0,067
131	0,026	128	0,090	91	0,114	116	0,147	6	0,229	25	0,044	78	0,096	16	0,058
32	0,026	56	0,083	32	0,075	1	0,119	126	0,225	36	0,040	130	0,088	111	0,058
25	0,020	29	0,073	65	0,067	36	0,116	42	0,195	127	0,034	65	0,083	46	0,057
110	0,020	7	0,072	119	0,067	136	0,114	110	0,184	6	0,027	70	0,082	17	0,053
6	0,020	115	0,067	83	0,066	70	0,093	67	0,111	109	0,019	6	0,077	91	0,046
15	0,020	67	0,064	115	0,060	76	0,073	91	0,091	130	0,016	115	0,072	70	0,038
62	0,020	129	0,060	76	0,059	126	0,059	131	0,085	139	0,016	42	0,070	41	0,035
16	0,016	42	0,058	16	0,057	42	0,059	29	0,080	42	0,015	110	0,064	78	0,034
42	0,016	5	0,057	139	0,048	38	0,055	94	0,079	67	0,013	112	0,062	44	0,034
54	0,016	27	0,056	41	0,047	129	0,054	27	0,038	94	0,010	36	0,058	2	0,026
1	0,016	15	0,051	15	0,033	134	0,046	78	0,026	131	0,010	48	0,055	132	0,026
36	0,000	8	0,049	9	0,029	110	0,045	9	0,026	1	0,008	55	0,045	1	0,024
38	0,000	16	0,046	36	0,027	41	0,040	119	0,025	19	0,008	129	0,042	9	0,023
41	0,000	10	0,045	27	0,020	65	0,036	36	0,022	27	0,007	83	0,041	19	0,017
55	0,000	126	0,037	129	0,015	139	0,034	136	0,020	72	0,007	46	0,038	3	0,017
109	0,000	134	0,034	107	0,014	109	0,031	106	0,019	83	0,007	67	0,035	25	0,017
126	0,000	110	0,025	138	0,008	78	0,031	1	0,015	78	0,004	126	0,026	36	0,014
128	0,000	3	0,023	109	0,006	72	0,027	130	0,015	46	0,004	2	0,024	55	0,014
134	0,000	55	0,021	20	0,006	27	0,026	129	0,013	136	0,004	5	0,024	117	0,014
138	0,000	78	0,019	1	0,000	2	0,023	32	0,012	138	0,003	76	0,022	101	0,014
78	0,000	35	0,015	6	0,000	107	0,022	76	0,012	48	0,003	4	0,020	94	0,012
139	0,000	14	0,015	67	0,000	104	0,020	72	0,011	7	0,003	27	0,019	131	0,012
2	0,000	58	0,015	78	0,000	32	0,019	127	0,011	129	0,002	32	0,018	6	0,012
19	0,000	107	0,015	108	0,000	64	0,016	83	0,011	9	0,000	3	0,018	127	0,012
46	0,000	130	0,015	2	0,000	121	0,012	109	0,011	15	0,000	131	0,015	129	0,006
48	0,000	20	0,011	19	0,000	15	0,010	107	0,010	116	0,000	94	0,015	15	0,006
72	0,000	21	0,008	46	0,000	89	0,009	3	0,010	32	0,000	109	0,014	138	0,006
107	0,000	31	0,008	48	0,000	5	0,007	139	0,009	2	0,000	139	0,014	114	0,006
130	0,000	40	0,008	72	0,000	23	0,007	46	0,007	107	0,000	24	0,014	80	0,000
3	0,000	43	0,008	130	0,000	135	0,007	2	0,006	3	0,000	15	0,014	32	0,000
5	0,000	37	0,007	3	0,000	6	0,006	24	0,004	5	0,000	7	0,012	67	0,000
29	0,000	109	0,006	5	0,000	67	0,006	121	0,004	4	0,000	119	0,011	107	0,000
91	0,000	41	0,005	106	0,000	46	0,004	63	0,003	24	0,000	19	0,010	130	0,000
106	0,000	83	0,004	127	0,000	49	0,003	125	0,002	49	0,000	49	0,008	5	0,000
127	0,000	24	0,004	136	0,000	63	0,003	58	0,002	63	0,000	9	0,006	136	0,000
136	0,000	23	0,004	4	0,000	19	0,001	5	0,001	68	0,000	107	0,005	4	0,000
4	0,000	26	0,004	7	0,000	48	0,001	4	0,001	121	0,000	39	0,004	7	0,000
7	0,000	28	0,004	24	0,000	4	0,001	49	0,001	20	0,000	121	0,004	24	0,000
24	0,000	65	0,003	49	0,000	39	0,001	35	0,001	23	0,000	127	0,004	49	0,000
49	0,000	36	0,003	63	0,000	112	0,001	117	0,001	35	0,000	123	0,004	63	0,000
63	0,000	138	0,002	68	0,000	123	0,001	15	0,001	37	0,000	63	0,002	121	0,000
68	0,000	139	0,000	121	0,000	47	0,001	138	0,001	39	0,000	89	0,002	20	0,000
121	0,000	19	0,000	23	0,000	108	0,000	19	0,001	58	0,000	138	0,001	23	0,000
20	0,000	46	0,000	35	0,000	3	0,000	68	0,001	64	0,000	68	0,001	35	0,000
23	0,000	48	0,000	37	0,000	106	0,000	7	0,000	89	0,000	37	0,001	37	0,000
35	0,000	72	0,000	39	0,000	127	0,000	20	0,000	112	0,000	132	0,001	39	0,000

>50
>20
10-20
5-10
1-5
0.5-1
0.1-0.5
0.05-0.1
0.01-0.05
0.005-0.01
<0.005

APPENDIX -2: Environmental taxa in biofilter biofilm.

	C2W3		C2W8		C2W13		WashI		C3W4		C3W8		C4W2		C5W10
158	2,430	187	3,246	187	2,861	187	5,244	233	1,998	150	3,109	248	11,392	233	11,712
64	1,719	153	1,267	153	2,338	45	3,187	64	1,497	64	1,869	158	5,917	187	6,087
190	0,879	158	1,012	55	1,364	153	1,819	45	1,429	62	1,404	190	3,303	236	5,997
232	0,643	64	0,836	150	0,862	72	0,939	187	1,360	218	1,187	237	2,007	64	2,824
153	0,626	55	0,550	158	0,681	55	0,835	218	0,884	187	1,067	153	1,456	158	1,665
187	0,380	237	0,528	45	0,599	123	0,681	27	0,827	153	0,812	187	1,289	153	1,251
55	0,368	51	0,274	64	0,542	158	0,536	150	0,737	233	0,466	152	1,134	50	1,035
159	0,344	70	0,236	224	0,519	64	0,384	158	0,673	158	0,450	45	1,030	21	0,840
50	0,194	138	0,216	124	0,505	190	0,293	123	0,564	159	0,410	64	1,022	124	0,808
45	0,174	45	0,212	237	0,421	218	0,265	72	0,543	124	0,345	191	0,980	19	0,671
224	0,158	123	0,199	218	0,388	232	0,259	153	0,508	45	0,324	50	0,768	188	0,486
222	0,152	42	0,128	72	0,321	159	0,248	159	0,461	190	0,271	21	0,717	237	0,476
72	0,114	78	0,121	138	0,226	237	0,235	230	0,309	50	0,178	159	0,539	159	0,440
174	0,110	157	0,115	236	0,193	150	0,226	182	0,260	237	0,177	123	0,346	283	0,378
236	0,109	190	0,113	159	0,190	224	0,219	138	0,252	224	0,160	72	0,286	224	0,368
78	0,097	50	0,112	50	0,179	51	0,215	237	0,252	182	0,120	113	0,273	27	0,350
280	0,097	310	0,096	51	0,150	135	0,187	50	0,179	27	0,107	124	0,263	193	0,299
70	0,094	182	0,096	123	0,147	154	0,163	54	0,171	70	0,106	145	0,262	113	0,277
237	0,091	159	0,083	162	0,114	138	0,155	124	0,165	230	0,077	54	0,256	240	0,246
29	0,074	72	0,075	29	0,105	124	0,137	135	0,158	72	0,076	150	0,250	138	0,234
218	0,071	150	0,068	41	0,103	78	0,093	236	0,146	123	0,067	19	0,238	306	0,231
144	0,059	38	0,067	154	0,091	70	0,077	19	0,140	236	0,063	51	0,218	42	0,167
192	0,035	236	0,062	30	0,072	227	0,075	21	0,139	21	0,056	218	0,190	45	0,165
253	0,032	124	0,060	182	0,072	162	0,072	44	0,133	51	0,054	236	0,154	245	0,125
205	0,029	62	0,045	40	0,057	19	0,068	190	0,087	19	0,050	138	0,142	182	0,095
156	0,026	167	0,039	156	0,055	256	0,065	224	0,083	44	0,048	182	0,130	10	0,089
193	0,026	192	0,038	135	0,045	71	0,064	113	0,079	113	0,036	267	0,112	218	0,084
154	0,020	171	0,030	157	0,043	236	0,055	2	0,068	54	0,034	70	0,090	38	0,082
246	0,020	241	0,029	167	0,034	66	0,053	78	0,064	138	0,032	2	0,082	18	0,073
40	0,016	135	0,029	78	0,031	41	0,052	221	0,062	156	0,020	251	0,081	114	0,072
47	0,016	170	0,026	222	0,028	188	0,046	167	0,061	204	0,017	41	0,068	204	0,070
163	0,016	19	0,026	19	0,027	98	0,044	235	0,051	175	0,013	146	0,066	131	0,068
221	0,016	29	0,025	190	0,022	283	0,044	154	0,037	69	0,013	18	0,063	230	0,063
230	0,016	154	0,024	230	0,022	233	0,039	204	0,037	162	0,013	245	0,057	164	0,059
243	0,016	43	0,023	306	0,020	167	0,039	162	0,036	283	0,012	276	0,055	78	0,052
256	0,016	218	0,020	204	0,019	204	0,032	62	0,034	235	0,010	135	0,052	162	0,052
18	0,010	178	0,020	221	0,019	182	0,032	38	0,034	285	0,010	204	0,045	150	0,047
42	0,010	156	0,016	235	0,019	21	0,030	70	0,025	295	0,010	235	0,041	55	0,041
51	0,010	144	0,014	276	0,019	50	0,025	119	0,025	57	0,008	233	0,041	144	0,038
58	0,010	205	0,013	139	0,015	40	0,024	205	0,024	55	0,008	62	0,038	206	0,035
235	0,010	142	0,012	65	0,013	230	0,023	51	0,022	2	0,007	167	0,036	192	0,031
1	0,000	305	0,012	227	0,013	111	0,023	188	0,022	18	0,007	281	0,035	222	0,026
2	0,000	283	0,012	188	0,013	142	0,022	42	0,016	135	0,007	230	0,033	246	0,023
3	0,000	306	0,011	18	0,008	229	0,022	156	0,016	144	0,007	239	0,033	2	0,020
4	0,000	230	0,010	38	0,008	240	0,021	246	0,016	154	0,007	295	0,032	190	0,018
5	0,000	253	0,007	70	0,006	306	0,019	222	0,015	243	0,006	119	0,032	305	0,017
6	0,000	233	0,007	71	0,006	65	0,019	18	0,014	267	0,006	178	0,031	135	0,014
7	0,000	172	0,006	142	0,006	278	0,017	232	0,013	80	0,005	224	0,031	154	0,014
8	0,000	162	0,006	178	0,006	235	0,016	194	0,012	98	0,005	188	0,029	169	0,014
9	0,000	40	0,005	192	0,006	192	0,015	139	0,011	222	0,005	78	0,028	238	0,014
10	0,000	232	0,005	305	0,006	243	0,014	245	0,010	65	0,004	162	0,027	256	0,014
11	0,000	224	0,005	1	0,000	246	0,014	65	0,010	167	0,004	27	0,025	71	0,012
12	0,000	30	0,004	2	0,000	30	0,013	55	0,009	188	0,004	136	0,023	174	0,012
13	0,000	54	0,004	3	0,000	221	0,012	227	0,009	221	0,004	144	0,023	267	0,012
14	0,000	4	0,003	4	0,000	194	0,010	178	0,008	241	0,004	44	0,022	310	0,012
15	0,000	222	0,003	5	0,000	253	0,010	239	0,008	245	0,004	210	0,022	29	0,009
16	0,000	269	0,003	6	0,000	241	0,009	142	0,007	246	0,004	114	0,021	72	0,009
17	0,000	58	0,003	7	0,000	69	0,009	71	0,007	116	0,003	238	0,021	134	0,009
19	0,000	146	0,003	8	0,000	295	0,009	29	0,006	174	0,003	203	0,020	178	0,009
20	0,000	163	0,003	9	0,000	54	0,007	295	0,006	227	0,003	131	0,018	194	0,009
21	0,000	21	0,002	10	0,000	42	0,007	243	0,006	282	0,003	205	0,018	205	0,009
22	0,000	44	0,002	11	0,000	43	0,007	276	0,006	30	0,002	222	0,017	253	0,009
23	0,000	264	0,002	12	0,000	52	0,007	306	0,006	58	0,002	29	0,016	287	0,009
24	0,000	27	0,002	13	0,000	193	0,007	199	0,006	304	0,002	192	0,015	301	0,009
25	0,000	46	0,002	14	0,000	207	0,007	41	0,005	310	0,002	20	0,014	40	0,006
26	0,000	221	0,002	15	0,000	228	0,007	256	0,005	1	0,000	240	0,013	62	0,006
27	0,000	246	0,002	16	0,000	248	0,007	137	0,004	3	0,000	30	0,011	85	0,006
28	0,000	248	0,002	17	0,000	282	0,007	69	0,004	4	0,000	259	0,011	89	0,006
30	0,000	1	0,000	20	0,000	157	0,006	310	0,004	5	0,000	69	0,011	139	0,006
31	0,000	2	0,000	21	0,000	215	0,006	269	0,004	6	0,000	38	0,011	157	0,006
32	0,000	3	0,000	22	0,000	16	0,004	277	0,003	7	0,000	65	0,009	170	0,006
33	0,000	5	0,000	23	0,000	29	0,003	144	0,003	8	0,000	174	0,009	243	0,006
34	0,000	6	0,000	24	0,000	296	0,003	173	0,003	9	0,000	194	0,009	1	0,000
35	0,000	7	0,000	25	0,000	305	0,003	283	0,003	10	0,000	148	0,009	3	0,000
36	0,000	8	0,000	26	0,000	18	0,001	1	0,003	11	0,000	306	0,008	4	0,000
37	0,000	9	0,000	27	0,000	35	0,001	66	0,003	12	0,000	175	0,008	5	0,000
38	0,000	10	0,000	28	0,000	38	0,001	157	0,002	13	0,000	268	0,007	6	0,000
39	0,000	11	0,000	31	0,000	102	0,001	20	0,002	14	0,000	170	0,007	7	0,000
41	0,000	12	0,000	32	0,000	139	0,001	278	0,002	15	0,000	310	0,007	8	0,000
43	0,000	13	0,000	33	0,000	143	0,001	241	0,002	16	0,000	149	0,006	9	0,000
44	0,000	14	0,000	34	0,000	156	0,001	253	0,002	17	0,000	253	0,006	11	0,000
46	0,000	15	0,000	35	0,000	174	0,001	58	0,001	20	0,000	154	0,006	12	0,000
48	0,000	16	0,000	36	0,000	259	0,001	102	0,001	22	0,000	202	0,006	13	0,000
49	0,000	17	0,000	37	0,000	276	0,001	145	0,001	23	0,000	283	0,006	14	0,000
52	0,000	18	0,000	39	0,000	1	0,000	146	0,001	24	0,000	40	0,005	15	0,000
53	0,000	20	0,000	42	0,000	2	0,000	280	0,001	25	0,000	256	0,005	16	0,000
54	0,000	22	0,000	43	0,000	3	0,000	286	0,001	26	0,000	243	0,005	17	0,000

>50
>20
10-20
5-10
1-5
0.5-1
0.1-0.5
0.05-0.1
0.01-0.05
0.005-0.01
<0.005

APPENDIX -3: Inoculums associated taxa in wall biofilm.

	C2W3		C2W8		C2W13		C3W4		C3W8		C4W2		C4W10		C5W2		C5W10
71	14,277	71	29,968	86	24,328	34	26,187	86	13,667	30	32,421	71	33,164	34	11,75	71	11,555
13	10,427	34	20,219	34	18,067	11	20,184	11	13,475	11	12,211	86	15,518	30	7,7161	66	5,4567
86	8,0191	11	13,373	71	15,902	71	12,247	34	13,124	71	10,766	12	10,95	96	7,3363	96	4,38
95	7,9296	96	6,6366	96	9,7611	96	7,0971	71	8,7863	86	7,7418	66	8,1702	71	6,3802	34	3,1027
96	4,6364	80	5,0001	11	5,2047	30	5,5896	30	8,4231	96	7,7281	34	3,9642	11	5,6727	86	2,9444
11	4,2483	86	3,0128	80	4,2262	13	3,2551	134	6,3106	34	3,3159	134	3,6742	85	4,9166	30	2,5902
133	2,945	18	3,0073	66	3,2465	38	2,7481	13	6,041	38	2,3379	96	2,6831	86	3,4109	12	2,5096
94	2,9151	13	1,7601	18	2,4471	22	2,1745	96	4,7657	13	2,0715	104	2,1513	13	2,3358	13	1,9198
80	2,8355	22	1,6401	56	0,8871	12	1,6275	108	2,0049	16	1,437	85	1,7646	22	2,3353	106	1,691
18	2,6167	66	1,3963	124	0,8374	86	1,5208	12	1,8786	12	1,3407	13	0,9911	12	2,2905	85	1,5862
99	2,6167	108	1,0574	95	0,7766	70	1,3074	65	1,6499	134	0,9349	133	0,7252	65	2,1476	22	1,2848
22	2,0595	38	1,0268	61	0,516	54	1,1206	66	1,5955	18	0,896	106	0,5318	124	1,9531	139	1,1452
34	2,0396	57	0,5086	22	0,4778	65	0,8805	57	1,3561	108	0,7163	30	0,4109	134	1,8162	38	0,8267
119	1,6615	120	0,4892	13	0,4669	111	0,707	22	1,1818	99	0,6247	54	0,3384	128	1,4927	18	0,7759
56	1,4625	12	0,4205	12	0,4521	57	0,627	85	1,0354	54	0,6114	116	0,3384	18	1,3438	78	0,676
116	1,2835	124	0,333	133	0,4106	18	0,4936	99	0,7917	22	0,6055	11	0,2901	120	1,284	11	0,6195
124	1,0546	99	0,1979	99	0,3951	134	0,1868	126	0,7225	55	0,6048	128	0,2901	110	0,9096	80	0,457
57	1,0447	111	0,1942	30	0,3281	108	0,1734	54	0,683	57	0,594	61	0,2417	108	0,7898	104	0,418
12	1,0148	30	0,1848	55	0,3051	56	0,1601	38	0,458	85	0,5177	57	0,2175	38	0,765	124	0,3952
111	0,8258	61	0,1841	120	0,2533	95	0,1467	18	0,4533	61	0,4956	99	0,2175	139	0,7162	108	0,3596
137	0,8059	119	0,1487	119	0,2265	42	0,1334	16	0,3561	41	0,4193	38	0,1934	57	0,7081	61	0,3413
17	0,5572	54	0,1448	57	0,2175	128	0,1334	42	0,2555	17	0,4013	124	0,1934	54	0,626	42	0,3097
25	0,4477	133	0,0946	128	0,1615	99	0,1201	104	0,2547	116	0,3668	56	0,1692	66	0,4701	134	0,2844
61	0,4378	55	0,0747	54	0,147	55	0,08	55	0,2406	95	0,3178	95	0,145	99	0,4097	54	0,2311
30	0,3781	15	0,072	111	0,1451	104	0,08	95	0,2087	130	0,292	108	0,1209	127	0,3886	133	0,2309
27	0,3482	95	0,0508	134	0,1253	67	0,0667	70	0,1995	133	0,1899	16	0,0967	136	0,3721	126	0,1594
120	0,3283	129	0,0421	110	0,1164	110	0,0667	111	0,1781	120	0,1862	111	0,0967	16	0,2887	65	0,1585
70	0,3184	27	0,0287	126	0,0966	16	0,0534	56	0,1363	111	0,1583	126	0,0967	70	0,281	16	0,1582
115	0,3184	131	0,0287	85	0,0826	29	0,0534	120	0,1346	80	0,1347	130	0,0967	133	0,2777	56	0,1512
85	0,2388	29	0,025	108	0,0826	124	0,0534	17	0,1078	66	0,1344	17	0,0725	56	0,2645	57	0,1436
76	0,2288	85	0,0216	131	0,0766	136	0,0534	61	0,0988	124	0,1176	22	0,0725	116	0,246	95	0,1179
104	0,189	17	0,0199	29	0,0539	80	0,04	67	0,0966	128	0,1077	139	0,0725	111	0,2293	27	0,1029
9	0,1791	70	0,0164	38	0,0512	133	0,04	128	0,0828	106	0,0878	18	0,0483	126	0,1931	64	0,0864
67	0,1791	115	0,0122	139	0,0416	1	0,0267	133	0,0786	70	0,0804	70	0,0483	55	0,1766	46	0,0718
109	0,1592	110	0,012	104	0,041	17	0,0267	41	0,0728	115	0,0701	115	0,0483	42	0,1634	128	0,0716
66	0,1492	134	0,0093	65	0,0348	66	0,0267	27	0,0686	67	0,0568	138	0,0483	61	0,1552	116	0,0704
131	0,1492	6	0,0088	27	0,0331	85	0,0267	124	0,0547	109	0,0472	1	0,0242	80	0,1547	91	0,0626
108	0,1293	42	0,0086	115	0,0244	126	0,0267	91	0,0542	83	0,0431	41	0,0242	95	0,1441	41	0,0557
6	0,1094	76	0,0086	32	0,0235	2	0,0133	116	0,044	136	0,0431	42	0,0242	17	0,1196	120	0,0555
38	0,1094	56	0,0083	1	0,02	6	0,0133	115	0,0428	48	0,0428	46	0,0242	104	0,1128	99	0,0477
54	0,1094	65	0,0081	6	0,0175	41	0,0133	6	0,0425	65	0,041	55	0,0242	41	0,094	109	0,032
32	0,0597	78	0,0079	130	0,0156	61	0,0133	80	0,0287	104	0,0372	83	0,0242	91	0,0663	70	0,0317
64	0,0398	62	0,0047	91	0,0104	116	0,0133	29	0,0282	64	0,0354	109	0,0242	115	0,0663	111	0,0315
36	0,0298	128	0,0047	16	0,0096	120	0,0133	110	0,0282	94	0,0236	110	0,0242	6	0,0611	132	0,0311
78	0,0199	130	0,0047	78	0,0096	127	0,0133	109	0,0209	56	0,0233	120	0,0242	67	0,0554	48	0,0237
107	0,0199	2	0,0041	83	0,0096	130	0,0133	32	0,0187	1	0,0214	2	0	106	0,0527	3	0,0155
3	0,0099	7	0,0041	94	0,0096	3	0	127	0,0087	46	0,0214	3	0	64	0,0519	72	0,0081
15	0,0099	94	0,0041	67	0,0052	4	0	131	0,0071	110	0,0196	4	0	130	0,0487	83	0,0081
39	0,0099	16	0,0039	76	0,0052	5	0	15	0,0065	72	0,0177	5	0	131	0,0487	112	0,0081
48	0,0099	32	0,0039	2	0,0044	7	0	1	0,0051	36	0,0155	6	0	36	0,0386	1	0,008
58	0,0099	67	0,0039	17	0,0044	8	0	48	0,0051	42	0,0155	7	0	44	0,0386	55	0,008
110	0,0099	126	0,0039	33	0,0044	9	0	89	0,0036	25	0,0137	8	0	94	0,0266	6	0,0078
129	0,0099	1	0	58	0,0044	10	0	119	0,0036	29	0,0137	9	0	72	0,0241	9	0,0078
1	0	3	0	62	0,0044	14	0	138	0,0036	76	0,0137	10	0	1	0,0221	17	0,0078
2	0	4	0	70	0,0044	15	0	72	0,0029	6	0,0078	14	0	76	0,0209	131	0,0078
4	0	5	0	109	0,0044	19	0	77	0,0029	19	0,0078	15	0	32	0,0193	2	0
5	0	8	0	136	0,0044	20	0	78	0,0029	62	0,0078	19	0	27	0,0156	4	0
7	0	9	0	3	0	21	0	139	0,0029	91	0,0078	20	0	29	0,0144	5	0
8	0	10	0	4	0	23	0	2	0	129	0,0078	21	0	109	0,012	7	0
10	0	14	0	5	0	24	0	3	0	32	0,0059	23	0	78	0,0108	8	0
14	0	19	0	7	0	25	0	4	0	139	0,0059	24	0	101	0,008	10	0
16	0	20	0	8	0	26	0	5	0	2	0	25	0	129	0,008	14	0
19	0	21	0	9	0	27	0	7	0	3	0	26	0	48	0,0048	15	0
20	0	23	0	10	0	28	0	8	0	4	0	27	0	83	0,0048	19	0
21	0	24	0	14	0	31	0	9	0	5	0	28	0	2	0,0036	20	0
23	0	25	0	15	0	32	0	10	0	7	0	29	0	15	0,0036	21	0
24	0	26	0	19	0	33	0	14	0	8	0	31	0	19	0,0024	23	0
26	0	28	0	20	0	35	0	19	0	9	0	32	0	121	0,0024	24	0
28	0	31	0	21	0	36	0	20	0	10	0	33	0	49	0,0012	25	0
29	0	33	0	23	0	37	0	21	0	14	0	35	0	119	0,0012	26	0
31	0	35	0	24	0	39	0	23	0	15	0	36	0	135	0,0012	28	0

- >50
- >20
- 10-20
- 5-10
- 1-5
- 0.5-1
- 0.1-0.5
- 0.05-0.1
- 0.01-0.05
- 0.005-0.01
- <0.005

APPENDIX -4: Environmental taxa in wall biofilm.

	C2W3		C2W8		C2W13		C3W4		C3W8		C4W2		C4W10		C5W2		C5W10
45	3,960	187	2,944	187	2,984	187	3,895	187	1,848	187	0,707	64	2,635	153	6,697	233	24,522
187	2,905	62	1,685	153	1,248	218	1,107	182	1,205	237	0,684	50	2,345	187	2,946	236	8,85
153	1,821	237	0,641	158	0,743	64	0,734	150	1,176	54	0,623	153	1,015	150	2,293	187	8,2359
64	1,482	158	0,491	55	0,640	123	0,667	218	0,917	153	0,570	159	0,822	158	1,727	50	1,4354
55	1,224	123	0,430	123	0,516	135	0,534	204	0,320	64	0,551	158	0,701	267	0,921	138	1,2328
50	1,065	64	0,405	218	0,480	44	0,494	64	0,309	224	0,524	45	0,604	19	0,896	153	1,019
158	1,065	138	0,369	64	0,304	182	0,454	138	0,265	182	0,446	237	0,435	21	0,724	159	0,756
190	0,637	153	0,311	124	0,242	138	0,320	237	0,187	45	0,354	190	0,411	167	0,697	124	0,6512
40	0,507	182	0,112	50	0,212	27	0,307	153	0,164	113	0,320	222	0,338	159	0,658	245	0,5414
78	0,378	70	0,098	236	0,207	237	0,240	205	0,159	236	0,269	236	0,266	64	0,653	64	0,5046
230	0,338	157	0,072	237	0,191	150	0,227	159	0,158	218	0,241	123	0,145	182	0,559	2	0,5011
159	0,298	310	0,071	138	0,174	62	0,147	158	0,132	158	0,236	21	0,121	218	0,538	158	0,4372
167	0,219	135	0,069	233	0,152	236	0,147	124	0,130	190	0,224	182	0,121	50	0,533	182	0,374
236	0,219	260	0,068	150	0,099	158	0,120	190	0,111	138	0,217	230	0,121	144	0,516	237	0,3426
253	0,169	159	0,067	224	0,097	159	0,120	44	0,103	21	0,209	240	0,121	224	0,423	193	0,2863
232	0,139	78	0,058	159	0,059	205	0,120	236	0,100	159	0,178	248	0,121	204	0,388	188	0,221
235	0,129	190	0,055	52	0,046	124	0,093	224	0,099	267	0,146	150	0,097	239	0,387	218	0,158
192	0,119	236	0,050	78	0,045	153	0,093	78	0,094	222	0,144	205	0,097	237	0,387	19	0,1495
218	0,109	171	0,046	182	0,043	224	0,080	27	0,091	19	0,131	221	0,097	190	0,383	78	0,1344
224	0,109	124	0,043	45	0,039	50	0,067	123	0,089	245	0,123	235	0,097	281	0,313	224	0,1194
237	0,099	50	0,041	222	0,035	78	0,053	135	0,083	72	0,117	243	0,097	222	0,307	114	0,1118
246	0,080	192	0,039	135	0,034	204	0,053	50	0,078	188	0,113	245	0,097	44	0,299	157	0,102
135	0,060	224	0,034	157	0,033	19	0,040	70	0,068	150	0,104	187	0,073	138	0,286	192	0,0872
222	0,060	29	0,032	154	0,024	45	0,040	54	0,065	221	0,084	191	0,073	135	0,203	113	0,0869
19	0,050	42	0,022	156	0,016	72	0,040	233	0,061	135	0,076	138	0,048	124	0,201	256	0,0864
62	0,050	218	0,021	71	0,016	157	0,027	167	0,061	204	0,063	204	0,048	123	0,192	27	0,0786
239	0,050	305	0,021	29	0,010	222	0,027	21	0,055	124	0,061	218	0,048	78	0,162	21	0,0642
280	0,050	222	0,020	30	0,010	29	0,013	222	0,053	192	0,055	8	0,024	45	0,147	204	0,0563
29	0,040	253	0,017	54	0,010	71	0,013	230	0,038	205	0,055	46	0,024	205	0,145	306	0,0553
72	0,040	205	0,017	167	0,010	114	0,013	19	0,038	203	0,051	55	0,024	85	0,141	222	0,0552
163	0,040	150	0,017	40	0,010	154	0,013	221	0,035	44	0,047	65	0,024	139	0,129	267	0,0552
221	0,040	44	0,013	72	0,010	175	0,013	235	0,035	123	0,045	70	0,024	54	0,126	42	0,0479
269	0,040	55	0,013	290	0,009	233	0,013	72	0,034	167	0,043	113	0,024	27	0,099	295	0,0479
281	0,040	19	0,013	62	0,005	264	0,013	113	0,029	50	0,041	114	0,024	245	0,067	87	0,0477
174	0,030	139	0,009	230	0,005	310	0,013	142	0,029	51	0,033	124	0,024	240	0,065	150	0,0398
193	0,030	30	0,008	69	0,005	1	0,000	188	0,024	18	0,031	145	0,024	18	0,053	18	0,0396
205	0,030	167	0,008	188	0,005	2	0,000	45	0,020	70	0,031	161	0,024	70	0,051	135	0,0396
306	0,030	144	0,008	205	0,005	3	0,000	85	0,014	42	0,027	164	0,024	113	0,050	305	0,0321
42	0,020	156	0,005	51	0,004	4	0,000	246	0,012	78	0,024	170	0,024	29	0,049	40	0,0319
70	0,020	175	0,005	144	0,004	5	0,000	171	0,009	181	0,023	175	0,024	62	0,048	162	0,0315
71	0,020	178	0,005	190	0,004	6	0,000	192	0,009	38	0,021	309	0,024	221	0,041	240	0,0315
142	0,020	232	0,005	1	0,000	7	0,000	178	0,009	174	0,018	1	0,000	72	0,040	275	0,0315
283	0,020	161	0,004	2	0,000	8	0,000	276	0,009	230	0,018	2	0,000	233	0,037	310	0,0313
4	0,010	174	0,004	3	0,000	9	0,000	80	0,008	233	0,018	3	0,000	114	0,036	246	0,0241
27	0,010	227	0,004	4	0,000	10	0,000	156	0,008	30	0,016	4	0,000	230	0,034	287	0,0236
65	0,010	251	0,004	5	0,000	11	0,000	145	0,006	157	0,016	5	0,000	40	0,034	85	0,0235
111	0,010	43	0,004	6	0,000	12	0,000	157	0,006	114	0,014	6	0,000	236	0,033	190	0,0235
113	0,010	45	0,004	7	0,000	13	0,000	18	0,005	119	0,014	7	0,000	306	0,033	29	0,016
139	0,010	72	0,004	8	0,000	14	0,000	38	0,005	131	0,014	9	0,000	188	0,028	55	0,016
140	0,010	154	0,004	9	0,000	15	0,000	267	0,005	243	0,014	10	0,000	203	0,025	134	0,016
144	0,010	230	0,004	10	0,000	16	0,000	41	0,004	246	0,014	11	0,000	235	0,022	230	0,016
154	0,010	1	0,000	11	0,000	17	0,000	42	0,004	253	0,014	12	0,000	51	0,019	205	0,016
156	0,010	2	0,000	12	0,000	18	0,000	51	0,004	85	0,012	13	0,000	287	0,016	164	0,0158
170	0,010	3	0,000	13	0,000	20	0,000	119	0,004	139	0,012	14	0,000	269	0,014	45	0,0157
172	0,010	4	0,000	14	0,000	21	0,000	169	0,004	154	0,012	15	0,000	10	0,008	86	0,0157
241	0,010	5	0,000	15	0,000	22	0,000	181	0,004	235	0,012	16	0,000	60	0,008	131	0,0157
1	0,000	6	0,000	16	0,000	23	0,000	256	0,004	307	0,012	17	0,000	69	0,008	171	0,0157
2	0,000	7	0,000	17	0,000	24	0,000	29	0,003	43	0,008	18	0,000	86	0,008	38	0,0155
3	0,000	8	0,000	18	0,000	25	0,000	65	0,003	178	0,008	19	0,000	156	0,008	206	0,0155
5	0,000	9	0,000	19	0,000	26	0,000	229	0,003	227	0,008	20	0,000	195	0,008	46	0,0081
6	0,000	10	0,000	20	0,000	28	0,000	305	0,003	241	0,008	22	0,000	295	0,008	143	0,0081
7	0,000	11	0,000	21	0,000	30	0,000	310	0,003	255	0,008	23	0,000	142	0,007	154	0,0081
8	0,000	12	0,000	22	0,000	31	0,000	1	0,000	276	0,008	24	0,000	271	0,006	156	0,0081
9	0,000	13	0,000	23	0,000	32	0,000	2	0,000	29	0,006	25	0,000	4	0,005	241	0,0081
10	0,000	14	0,000	24	0,000	33	0,000	3	0,000	66	0,006	26	0,000	71	0,005	210	0,008
11	0,000	15	0,000	25	0,000	34	0,000	4	0,000	82	0,006	27	0,000	137	0,005	239	0,008
12	0,000	16	0,000	26	0,000	35	0,000	5	0,000	142	0,006	28	0,000	157	0,005	248	0,008
13	0,000	17	0,000	27	0,000	36	0,000	6	0,000	144	0,006	29	0,000	191	0,005	309	0,008
14	0,000	18	0,000	28	0,000	37	0,000	7	0,000	239	0,006	30	0,000	192	0,005	10	0,0078
15	0,000	20	0,000	31	0,000	38	0,000	8	0,000	240	0,006	31	0,000	246	0,005	144	0,0078
16	0,000	21	0,000	32	0,000	39	0,000	9	0,000	264	0,006	32	0,000	310	0,005	191	0,0078
17	0,000	22	0,000	33	0,000	40	0,000	10	0,000	295	0,006	33	0,000	30	0,004	258	0,0078
18	0,000	23	0,000	34	0,000	41	0,000	11	0,000	1	0,000	34	0,000	178	0,004	307	0,0078
20	0,000	24	0,000	35	0,000	42	0,000	12	0,000	2	0,000	35	0,000	181	0,004	1	0
21	0,000	25	0,000	36	0,000	43	0,000	13	0,000	3	0,000	36	0,000	241	0,004	3	0
22	0,000	26	0,000	37	0,000	46	0,000	14	0,000	4	0,000	37	0,000	251	0,004	4	0
23	0,000	27	0,000	38	0,000	47	0,000	15	0,000	5	0,000	38	0,000	256	0,004	5	0
24	0,000	28	0,000	39	0,000	48	0,000	16	0,000	6	0,000	39	0,000	259	0,004	6	0
25	0,000	31	0,000	41	0,000	49	0,000	17	0,000	7	0,000	40	0,000	20	0,002	7	0
26	0,000	32	0,000	42	0,000	51	0,000	20	0,000	8	0,000	41	0,000	169	0,002	8	0
28	0,000	33	0,000	43	0,000	52	0,000	22	0,000	9	0,000	42	0,000	253	0,002	9	0
30	0,000	34	0,000	44	0,000	53	0,000	23	0,000	10	0,000	43	0,000	265	0,002	11	0
31	0,000	35	0,000	46	0,000	54	0,000	24	0,000	11	0,000	44	0,000	276	0,002	12	0
32	0,000	36	0,000	47	0,000	55	0,000	25	0,000	12	0,000	47	0,000	280	0,002	13	0
33	0,000	37	0,000	48	0,000	56	0,000	26	0,000	13	0,000	48	0,000	305	0,002	14	0
34	0,000	38	0,000	49	0,000	57	0,000	28	0,000	14	0,000	49	0,000	2	0,001	15	0
35	0,000	39	0,000	53	0,000	58	0,000	30	0,000	15	0,000	51	0,000	38	0,001	16	0
36	0,000	40	0,000	56	0,000	59	0,000	31	0,000	16	0,000	52	0,000	87	0,001	17	0
37	0,000	41	0,000	57	0,000	60	0,000	32	0,000	17	0,000	53	0,000	146	0,001	20	0
38	0,000	46	0,000	58	0,000	61	0,000	33	0,000	20	0,000	54	0,000	164	0,001	22	0
39	0,000	47	0,000	59	0,000	63	0,000	34	0,000	22	0,000	56	0,000	171	0,001	23	0
41	0,000	48	0,000	60	0,000	65	0,000	35	0,000	23	0,000	57	0,000	275	0,001	24	0
43	0,000	49	0,000	61	0,000	66	0,000	36	0,000	24	0,000	58	0,000	285	0,001	25	0

- >50
- >20
- 10-20
- 5-10
- 1-5
- 0.5-1
- 0.1-0.5
- 0.05-0.1
- 0.01-0.05
- 0.005-0.01
- <0.005

APPENDIX -5: Inoculums associated taxa in water.

	C2W3		C2W8		C2W13		C3W4		C3W8		C4W2		C4W10		C5W2		C5W10
95	88,226	95	89,233	95	60,063	95	96,254	109	27,359	95	84,144	95	35,120	71	77,931	95	73,208
18	1,345	12	1,561	138	16,240	85	0,253	71	8,062	71	1,969	12	7,549	85	4,936	109	19,424
11	1,135	85	0,667	57	1,667	96	0,230	108	8,031	12	1,672	13	3,412	34	3,317	104	0,311
13	0,985	133	0,447	109	1,634	124	0,127	65	6,844	109	0,799	71	3,345	12	1,234	139	0,310
71	0,967	99	0,425	124	1,528	6	0,121	134	5,276	124	0,503	108	3,067	124	1,011	71	0,268
94	0,581	6	0,396	120	1,015	71	0,121	95	5,005	108	0,487	34	2,897	44	0,900	12	0,250
22	0,523	57	0,269	71	0,965	30	0,112	12	2,801	34	0,280	124	2,305	78	0,769	34	0,190
99	0,429	120	0,208	12	0,951	86	0,098	85	2,367	99	0,265	86	2,268	95	0,627	65	0,169
12	0,353	124	0,207	56	0,933	34	0,095	13	2,025	64	0,254	99	1,760	13	0,526	124	0,118
104	0,335	80	0,199	34	0,926	104	0,086	96	1,800	30	0,180	104	1,204	30	0,303	120	0,118
137	0,246	71	0,197	85	0,738	54	0,046	34	1,575	18	0,148	134	0,859	120	0,243	13	0,093
86	0,241	32	0,182	54	0,701	109	0,043	127	1,226	104	0,132	85	0,817	133	0,162	126	0,082
80	0,226	22	0,167	18	0,616	12	0,040	30	1,001	85	0,111	139	0,726	6	0,121	85	0,081
96	0,216	61	0,150	131	0,479	99	0,037	126	0,916	86	0,090	66	0,635	96	0,121	1	0,074
17	0,147	34	0,138	61	0,444	127	0,037	124	0,621	13	0,085	96	0,569	86	0,111	96	0,070
133	0,147	86	0,118	22	0,389	13	0,035	66	0,574	61	0,085	109	0,569	109	0,111	80	0,070
34	0,135	54	0,078	99	0,364	65	0,026	22	0,551	129	0,085	17	0,538	57	0,101	86	0,055
56	0,086	18	0,075	13	0,259	108	0,026	70	0,535	17	0,079	120	0,423	80	0,091	134	0,051
119	0,086	15	0,067	30	0,200	110	0,026	86	0,388	54	0,074	130	0,387	65	0,081	132	0,048
85	0,079	67	0,063	86	0,195	126	0,026	120	0,341	38	0,069	57	0,351	127	0,081	6	0,040
124	0,063	111	0,063	32	0,173	57	0,020	17	0,326	76	0,069	128	0,351	139	0,081	108	0,039
115	0,038	38	0,062	139	0,169	61	0,020	6	0,318	96	0,069	133	0,333	56	0,051	133	0,037
25	0,036	13	0,057	128	0,165	111	0,020	57	0,310	120	0,064	106	0,315	108	0,051	110	0,037
70	0,036	129	0,052	110	0,163	134	0,020	18	0,272	136	0,064	54	0,206	134	0,051	30	0,036
116	0,033	11	0,037	111	0,149	17	0,017	111	0,264	57	0,053	111	0,200	16	0,040	66	0,032
57	0,030	128	0,036	130	0,137	18	0,017	38	0,233	139	0,053	61	0,194	18	0,040	57	0,029
111	0,025	66	0,027	133	0,128	22	0,012	99	0,209	6	0,048	30	0,157	19	0,040	11	0,029
61	0,018	104	0,022	29	0,111	80	0,012	104	0,202	22	0,042	126	0,145	99	0,040	106	0,027
30	0,015	27	0,021	94	0,092	56	0,009	11	0,194	80	0,042	83	0,139	116	0,040	61	0,027
108	0,015	126	0,018	104	0,088	67	0,009	56	0,194	83	0,042	16	0,127	11	0,030	111	0,025
66	0,013	138	0,015	15	0,085	120	0,009	61	0,186	27	0,037	70	0,091	106	0,030	18	0,021
67	0,013	131	0,012	80	0,084	130	0,009	55	0,178	134	0,037	18	0,085	111	0,030	22	0,021
27	0,010	29	0,011	6	0,082	66	0,006	54	0,171	106	0,026	19	0,073	17	0,020	17	0,016
32	0,010	119	0,010	11	0,067	70	0,006	128	0,155	133	0,026	6	0,067	22	0,020	78	0,015
120	0,010	110	0,009	27	0,066	115	0,006	133	0,140	70	0,021	15	0,067	110	0,020	15	0,013
109	0,008	116	0,009	126	0,059	133	0,006	83	0,124	126	0,021	116	0,060	126	0,020	99	0,012
110	0,008	130	0,008	55	0,055	16	0,003	110	0,124	16	0,016	56	0,054	130	0,020	54	0,011
130	0,008	17	0,008	91	0,052	27	0,003	130	0,101	78	0,016	76	0,030	1	0,010	27	0,009
9	0,005	30	0,007	65	0,045	38	0,003	78	0,078	11	0,011	127	0,030	38	0,010	138	0,008
76	0,005	56	0,006	76	0,041	41	0,003	138	0,078	128	0,011	65	0,024	42	0,010	64	0,008
78	0,005	1	0,005	66	0,037	42	0,003	15	0,062	32	0,005	112	0,024	46	0,010	42	0,007
83	0,005	83	0,005	67	0,031	78	0,003	115	0,062	36	0,005	114	0,024	54	0,010	56	0,006
1	0,003	78	0,004	17	0,030	83	0,003	16	0,054	46	0,005	22	0,018	61	0,010	116	0,006
6	0,003	137	0,003	70	0,029	89	0,003	80	0,054	56	0,005	38	0,018	62	0,010	70	0,006
35	0,003	58	0,003	83	0,026	107	0,003	125	0,047	111	0,005	37	0,012	66	0,010	72	0,006
42	0,003	76	0,003	123	0,026	116	0,003	27	0,039	116	0,005	41	0,012	91	0,010	3	0,006
54	0,003	96	0,003	36	0,025	139	0,003	37	0,039	130	0,005	55	0,012	101	0,010	55	0,005
131	0,003	89	0,002	115	0,023	1	0,000	67	0,039	138	0,005	94	0,012	128	0,010	2	0,005
134	0,003	2	0,002	78	0,021	2	0,000	62	0,031	1	0,000	110	0,012	129	0,010	16	0,005
138	0,003	107	0,002	134	0,019	3	0,000	137	0,031	2	0,000	2	0,006	2	0,000	38	0,005
2	0,000	9	0,002	96	0,016	4	0,000	82	0,023	3	0,000	11	0,006	3	0,000	128	0,004
3	0,000	36	0,002	129	0,015	5	0,000	106	0,023	4	0,000	48	0,006	4	0,000	5	0,003
4	0,000	94	0,002	16	0,015	7	0,000	116	0,023	5	0,000	80	0,006	5	0,000	8	0,003
5	0,000	7	0,001	25	0,015	8	0,000	123	0,023	7	0,000	119	0,006	7	0,000	25	0,003
7	0,000	109	0,001	107	0,012	9	0,000	139	0,023	8	0,000	129	0,006	8	0,000	36	0,003
8	0,000	3	0,001	58	0,012	10	0,000	7	0,016	9	0,000	131	0,006	9	0,000	68	0,003
10	0,000	4	0,001	1	0,011	11	0,000	19	0,016	10	0,000	132	0,006	10	0,000	94	0,003
14	0,000	44	0,001	19	0,011	14	0,000	41	0,016	14	0,000	135	0,006	14	0,000	101	0,003
15	0,000	62	0,001	116	0,008	15	0,000	42	0,016	15	0,000	1	0,000	15	0,000	67	0,003
16	0,000	115	0,001	38	0,007	19	0,000	46	0,016	19	0,000	3	0,000	20	0,000	83	0,003
19	0,000	127	0,001	41	0,007	20	0,000	29	0,008	20	0,000	4	0,000	21	0,000	9	0,002
20	0,000	135	0,001	89	0,007	21	0,000	48	0,008	21	0,000	5	0,000	23	0,000	10	0,002
21	0,000	5	0,000	2	0,005	23	0,000	53	0,008	23	0,000	7	0,000	24	0,000	14	0,002
23	0,000	8	0,000	46	0,005	24	0,000	72	0,008	24	0,000	8	0,000	25	0,000	23	0,002
24	0,000	10	0,000	42	0,004	25	0,000	77	0,008	25	0,000	9	0,000	26	0,000	32	0,002
26	0,000	14	0,000	37	0,004	26	0,000	91	0,008	26	0,000	10	0,000	27	0,000	49	0,002
28	0,000	16	0,000	62	0,004	28	0,000	94	0,008	28	0,000	14	0,000	28	0,000	62	0,002
29	0,000	19	0,000	9	0,003	29	0,000	129	0,008	29	0,000	20	0,000	29	0,000	73	0,002
31	0,000	20	0,000	64	0,003	31	0,000	136	0,008	31	0,000	21	0,000	31	0,000	89	0,002
33	0,000	21	0,000	48	0,003	32	0,000	1	0,000	33	0,000	23	0,000	32	0,000	91	0,002
36	0,000	23	0,000	106	0,003	33	0,000	2	0,000	35	0,000	24	0,000	33	0,000	114	0,002
37	0,000	24	0,000	118	0,003	35	0,000	3	0,000	37	0,000	25	0,000	35	0,000	129	0,002
38	0,000	25	0,000	3	0,001	36	0,000	4	0,000	39	0,000	26	0,000	36	0,000	130	0,002
39	0,000	26	0,000	4	0,001	37	0,000	5	0,000	40	0,000	27	0,000	37	0,000	7	0,001
40	0,000	28	0,000	7	0,001	39	0,000	8	0,000	41	0,000	28	0,000	39	0,000	19	0,001
41	0,000	31	0,000	50	0,001	40	0,000	9	0,000	42	0,000	29	0,000	40	0,000	48	0,001
43	0,000	33	0,000	121	0,001	43	0,000	10	0,000	43	0,000	31	0,000	41	0,000	63	0,001
44	0,000	35	0,000	135	0,001	44	0,000	14	0,000	44	0,000	32	0,000	43	0,000	112	0,001
45	0,000	37	0,000	136	0,001	45	0,000	20	0,000	45	0,000	33	0,000	45	0,000	4	0,000

>50
>20
10-20
5-10
1-5
0.5-1
0.1-0.5
0.05-0.1
0.01-0.05
0.005-0.01
<0.005

APPENDIX -6: Environmental taxa in water.

	C2W3		C2W8		C2W13		C3W4		C3W8		C4W2		C4W10		C5W2		C5W10
232	0,828	190	1,847	256	2,959	27	0,622	243	1,816	21	1,392	230	7,368	60	1,376	233	1,545
244	0,536	187	0,622	232	0,492	232	0,392	150	1,466	232	1,085	190	5,928	281	0,799	232	0,628
45	0,269	232	0,460	150	0,401	150	0,141	187	1,304	70	0,783	153	4,162	21	0,738	21	0,567
64	0,236	157	0,212	55	0,332	187	0,115	70	0,970	244	0,556	240	1,204	187	0,475	187	0,198
190	0,208	42	0,183	187	0,314	230	0,089	190	0,900	158	0,492	158	0,968	230	0,465	64	0,195
187	0,170	55	0,182	153	0,282	233	0,086	227	0,861	243	0,392	64	0,919	158	0,364	236	0,149
55	0,142	38	0,164	66	0,265	244	0,040	64	0,854	187	0,201	191	0,817	237	0,303	27	0,084
158	0,112	153	0,162	21	0,211	64	0,032	229	0,799	283	0,201	306	0,653	27	0,273	256	0,079
70	0,076	64	0,131	64	0,143	204	0,032	283	0,691	237	0,180	237	0,502	153	0,162	29	0,076
153	0,074	227	0,083	135	0,134	138	0,026	218	0,636	227	0,169	174	0,466	190	0,142	158	0,064
224	0,033	138	0,050	230	0,122	54	0,023	167	0,628	64	0,148	21	0,442	19	0,131	193	0,061
230	0,030	181	0,041	224	0,116	218	0,023	21	0,590	150	0,122	123	0,442	204	0,131	153	0,057
243	0,030	150	0,035	158	0,114	229	0,023	230	0,535	190	0,101	232	0,436	283	0,081	55	0,057
50	0,025	124	0,035	276	0,107	21	0,020	27	0,411	230	0,095	170	0,308	64	0,071	70	0,047
174	0,023	170	0,033	124	0,095	70	0,017	204	0,372	153	0,090	27	0,260	241	0,071	38	0,042
283	0,023	51	0,030	41	0,092	153	0,017	153	0,326	276	0,085	150	0,248	29	0,061	283	0,035
178	0,020	29	0,023	227	0,078	243	0,017	50	0,264	19	0,079	146	0,188	239	0,061	240	0,034
222	0,015	70	0,019	138	0,074	62	0,014	116	0,225	136	0,074	227	0,169	124	0,040	230	0,024
29	0,013	230	0,017	72	0,063	264	0,014	267	0,209	167	0,074	145	0,157	144	0,040	57	0,022
40	0,013	123	0,016	190	0,049	19	0,012	256	0,202	181	0,074	229	0,157	236	0,040	50	0,019
72	0,013	241	0,015	157	0,048	158	0,012	233	0,194	27	0,064	50	0,151	280	0,040	171	0,019
218	0,013	229	0,015	123	0,044	8	0,009	224	0,186	2	0,058	256	0,145	224	0,030	178	0,016
42	0,010	175	0,012	93	0,041	72	0,009	124	0,140	62	0,058	236	0,121	233	0,030	275	0,016
56	0,010	71	0,012	159	0,034	157	0,009	178	0,140	253	0,058	204	0,115	2	0,020	18	0,015
71	0,010	310	0,011	45	0,033	227	0,009	19	0,132	178	0,053	248	0,115	18	0,020	190	0,015
78	0,010	19	0,011	204	0,033	45	0,006	54	0,124	204	0,053	224	0,097	38	0,020	237	0,014
159	0,010	305	0,010	218	0,030	55	0,006	182	0,124	240	0,053	187	0,091	50	0,020	241	0,014
144	0,008	167	0,009	19	0,029	111	0,006	158	0,116	18	0,048	243	0,091	70	0,020	162	0,013
236	0,008	171	0,009	50	0,027	124	0,006	282	0,116	251	0,048	38	0,085	89	0,020	19	0,012
253	0,008	116	0,009	30	0,027	135	0,006	80	0,101	45	0,042	167	0,079	111	0,020	170	0,012
280	0,008	192	0,008	42	0,021	159	0,006	111	0,101	229	0,042	238	0,079	159	0,020	124	0,011
281	0,008	238	0,008	162	0,019	161	0,006	119	0,101	119	0,037	43	0,073	171	0,020	150	0,011
18	0,005	162	0,007	29	0,019	167	0,006	159	0,101	274	0,037	152	0,073	193	0,020	144	0,010
156	0,005	62	0,007	27	0,019	182	0,006	8	0,085	123	0,032	29	0,067	240	0,020	191	0,010
163	0,005	159	0,007	178	0,018	280	0,006	44	0,078	124	0,032	55	0,067	256	0,020	244	0,010
192	0,005	163	0,007	78	0,018	281	0,006	45	0,078	256	0,032	159	0,067	259	0,020	138	0,009
221	0,005	158	0,006	167	0,014	298	0,006	298	0,078	144	0,026	277	0,067	268	0,020	188	0,009
225	0,005	237	0,006	243	0,014	2	0,003	40	0,070	267	0,026	36	0,042	306	0,020	224	0,009
227	0,005	30	0,005	277	0,014	29	0,003	235	0,070	268	0,026	70	0,042	6	0,010	85	0,009
235	0,005	50	0,005	38	0,014	38	0,003	138	0,062	308	0,026	19	0,036	40	0,010	238	0,009
256	0,005	154	0,005	154	0,014	50	0,003	263	0,062	113	0,021	45	0,036	42	0,010	42	0,009
19	0,003	36	0,004	194	0,012	89	0,003	270	0,062	192	0,021	222	0,036	54	0,010	280	0,009
20	0,003	72	0,004	51	0,011	119	0,003	29	0,054	235	0,021	235	0,036	71	0,010	84	0,008
30	0,003	4	0,004	71	0,011	123	0,003	145	0,054	277	0,021	135	0,030	84	0,010	204	0,008
38	0,003	45	0,004	192	0,011	133	0,003	237	0,054	281	0,021	164	0,030	87	0,010	2	0,007
43	0,003	33	0,003	188	0,010	162	0,003	241	0,054	298	0,021	178	0,030	136	0,010	265	0,007
47	0,003	178	0,003	111	0,008	178	0,003	268	0,054	29	0,016	192	0,030	138	0,010	157	0,006
99	0,003	306	0,003	306	0,008	186	0,003	269	0,054	50	0,016	205	0,030	150	0,010	62	0,006
105	0,003	41	0,003	4	0,008	188	0,003	296	0,054	54	0,016	245	0,030	164	0,010	245	0,006
142	0,003	111	0,003	40	0,008	190	0,003	304	0,054	82	0,016	111	0,024	178	0,010	306	0,006
162	0,003	174	0,003	265		215	0,003	41	0,047	111	0,016	142	0,024	185	0,010	40	0,006
173	0,003	182	0,003	235	0,007	235	0,003	98	0,047	159	0,016	212	0,024	191	0,010	72	0,005
182	0,003	281	0,003	142	0,007	241	0,003	188	0,047	38	0,011	297	0,024	202	0,010	113	0,005
205	0,003	98	0,002	283	0,007	248	0,003	238	0,047	41	0,011	60	0,018	205	0,010	131	0,005
237	0,003	215	0,002	170	0,007	256	0,003	264	0,047	51	0,011	114	0,018	227	0,010	243	0,005
264	0,003	283	0,002	171	0,007	273	0,003	277	0,047	72	0,011	139	0,018	229	0,010	258	0,005
282	0,003	27	0,002	182	0,007	277	0,003	42	0,039	98	0,011	171	0,018	245	0,010	45	0,004
298	0,003	137	0,002	270	0,007	284	0,003	99	0,039	162	0,011	265	0,018	258	0,010	175	0,004
306	0,003	256	0,002	237	0,006	1	0,000	113	0,039	173	0,011	9	0,012	263	0,010	8	0,003
1	0,000	278	0,002	238	0,006	3	0,000	232	0,039	203	0,011	30	0,012	264	0,010	66	0,003
2	0,000	43	0,002	61	0,005	4	0,000	236	0,039	225	0,011	98	0,012	265	0,010	89	0,003
3	0,000	135	0,002	137	0,005	5	0,000	285	0,039	241	0,011	113	0,012	293	0,010	167	0,003
4	0,000	236	0,002	43	0,004	6	0,000	31	0,031	265	0,011	119	0,012	1	0,000	174	0,003
5	0,000	243	0,002	18	0,004	7	0,000	33	0,031	296	0,011	175	0,012	3	0,000	192	0,003
6	0,000	91	0,001	70	0,004	9	0,000	57	0,031	30	0,005	202	0,012	4	0,000	218	0,003
7	0,000	142	0,001	145	0,004	10	0,000	108	0,031	49	0,005	228	0,012	5	0,000	242	0,003
8	0,000	242	0,001	193	0,004	11	0,000	161	0,031	78	0,005	263	0,012	7	0,000	268	0,003
9	0,000	248	0,001	221	0,004	12	0,000	215	0,031	108	0,005	267	0,012	8	0,000	152	0,003
10	0,000	251	0,001	222	0,004	13	0,000	225	0,031	131	0,005	278	0,012	9	0,000	267	0,003
11	0,000	298	0,001	241	0,004	14	0,000	18	0,023	145	0,005	2	0,006	10	0,000	26	0,002
12	0,000	35	0,001	244	0,004	15	0,000	55	0,023	154	0,005	6	0,006	11	0,000	31	0,002
13	0,000	119	0,001	263	0,004	16	0,000	62	0,023	157	0,005	58	0,006	12	0,000	54	0,002
14	0,000	139	0,001	144	0,003	17	0,000	89	0,023	176	0,005	71	0,006	13	0,000	78	0,002
15	0,000	144	0,001	296	0,003	18	0,000	123	0,023	193	0,005	72	0,006	14	0,000	87	0,002
16	0,000	145	0,001	82	0,003	20	0,000	170	0,023	202	0,005	85	0,006	15	0,000	123	0,002
17	0,000	172	0,001	119	0,003	22	0,000	175	0,023	210	0,005	96	0,006	16	0,000	134	0,002
21	0,000	253	0,001	172	0,003	23	0,000	240	0,023	224	0,005	101	0,006	17	0,000	135	0,002
22	0,000	296	0,001	225	0,003	24	0,000	280	0,023	233	0,005	108	0,006	20	0,000	139	0,002
23	0,000	304	0,001	236	0,003	25	0,000	281	0,023	275	0,005	116	0,006	22	0,000	159	0,002
24	0,000	1	0,000	240	0,003	26	0,000	38	0,016	284	0,005	118	0,006	23	0,000	161	0,002
25	0,000	2	0,000	242	0,003	28	0,000	84	0,016	1	0,000	162	0,006	24	0,000	173	0,002
26	0,000	3	0,000	298	0,003	30	0,000	112	0,016	3	0,000	173	0,006	25	0,000	198	0,002
27	0,000	5	0,000	16	0,001	31	0,000	203	0,016	4	0,000	216	0,006	26	0,000	227	0,002
28	0,000	6	0,000	46	0,001	32	0,000	222	0,016	5	0,000	218	0,006	28	0,000	264	0,002
31	0,000	7	0,000	73	0,001	33	0,000	228	0,016	6	0,000	253	0,006	30	0,000	272	0,002
32	0,000	8	0,000	81	0,001	34	0,000	231	0,016	7	0,000	264	0,006	31	0,000	277	0,002
33	0,000	9	0,000	113	0,001	35	0,000	245	0,016	8	0,000	268	0,006	32	0,000	292	0,002
34	0,000	10	0,000	141	0,001	36	0,000	275	0,016	9	0,000	281	0,006	33	0,000	16	0,001
35	0,000	11	0,000	173	0,001	37	0,000	284	0,016	10	0,000	282	0,006	34	0,000	145	0,001
36	0,000	12	0,000	174	0,001	39	0,000	306	0,016	11	0,000	283	0,006	35	0,000	229	0,001
37	0,000	13	0,000	215	0,001	40	0,000	2	0,008	12	0,000	294	0,006	36	0,000	281	0,001
39	0,000	14	0,000	228	0,001	41	0,000	5	0,008	13	0,000	296	0,006	37	0,000	310	0,001
41	0,000	15	0,000	253	0,001	42	0,000	32	0,008	14	0,000	298	0,006	39	0,000	1	0,000
44	0,000	16	0,000	261	0,001	43	0,000	36	0,008	15	0,000	307	0,006	41	0,000	3	0,000
46	0,000	17	0,000	267	0,001	44	0,000	66	0,008	16	0,000	1	0,000	43	0,000	4	0,000
48	0,000	18	0,000	269	0,001	46	0,000	88	0,008	17	0,000	3	0,000	44	0,000	5	0,000
49	0,000	20	0,000	271	0,001	47	0,000	110	0,008	20	0,000	4	0,000	45	0,000	6	0,000
51	0,000	21	0,000	282	0,001	48	0,000	135	0,008	22	0,000	5	0,000	46	0,000	7	0,000
52	0,000	22	0,000	310	0,001	49	0,000	136	0,008	23	0,000	7	0,000	47	0,000	9	0,000
53	0,000	23	0,000	1	0,000	51	0,000	139	0,008	24	0,000	8	0,000	48	0,000	10	0,000
54	0,000	24	0,000	2	0,000	52	0,000	144	0,008	25	0,000	10	0,000	49	0,000	11	0,000
57	0,000	25	0,000	3	0,000	53	0,000	149	0,008	26	0,000	11	0,000	51	0,000	12	0,000
58	0,000	26	0,000	5	0,000	56	0,000	176	0,008	28	0,000	12	0,000	52	0,000	13	0,000
59	0,000	28	0,000	6	0,000	57	0,000	194	0,008	31	0,000	13	0,000	53	0,000	14	0,000
60	0,000	31	0,000	7	0,000	58	0,000	202	0,008	32	0,000	14	0,000	55	0,000	15	0,000
61	0,000	32	0,000	8	0,000	59	0,000	207	0,008	33	0,000	15	0,000	56	0,000	17	0,000
62	0,000	34	0,000	9	0,000	60	0,000	244	0,008	34	0,000	16	0,000	57	0,000	20	0,000
63	0,000	37	0,000	10	0,000	61	0,000	251	0,008	35	0,000	17	0,000	58	0,000	22	0,000
65	0,000	39	0,000	11	0,000	63	0,000	272	0,008	36	0,000	18	0,000	59	0,000	23	0,000
66	0,000	40	0,000	12	0,000	65	0,000	273	0,008	37	0,000	20	0,000	61	0,000	24	0,000
67	0,000	44	0,000	13	0,000	66	0,000	297	0,008	39	0,000	22	0,000	62	0,000	25	0,000
68	0,000	46	0,000	14	0,000	67	0,000	303	0,008	40	0,000	23	0,000	63	0,000	28	0,000
69	0,000	47	0,000	15	0,000	68	0,000	310	0,008	42	0,000	24	0,000	65	0,000	30	0,000

>50
>20
10-20
5-10
1-5
0.5-1
0.1-0.5
0.05-0.1
0.01-0.05
0.005-0.01
<0.005

APPENDIX -7: Inoculums associated taxa in fish skin wounds.

C2W3		C2W8		C2W13		C3W4	
11	1,127	95	17,634	104	0,073	34	0,023
95	0,336	11	2,870	95	0,036	12	0,009
71	0,164	71	1,949	85	0,032	1	0,005
18	0,101	99	0,830	12	0,024	22	0,005
104	0,090	34	0,646	34	0,024	95	0,005
13	0,082	104	0,607	71	0,024	99	0,005
17	0,078	83	0,496	65	0,016	104	0,005
12	0,060	120	0,473	61	0,012	108	0,005
99	0,056	12	0,426	99	0,012	109	0,005
96	0,052	139	0,423	18	0,008	2	0,000
1	0,049	86	0,420	120	0,008	3	0,000
94	0,045	2	0,383	1	0,004	4	0,000
22	0,037	109	0,290	25	0,004	5	0,000
34	0,022	108	0,230	30	0,004	6	0,000
85	0,022	96	0,223	56	0,004	7	0,000
137	0,022	1	0,193	86	0,004	8	0,000
25	0,011	61	0,190	106	0,004	9	0,000
111	0,011	38	0,180	108	0,004	10	0,000
138	0,011	133	0,140	126	0,004	11	0,000
56	0,007	134	0,140	138	0,004	13	0,000
65	0,007	70	0,137	2	0,000	14	0,000
70	0,007	85	0,137	3	0,000	15	0,000
86	0,007	3	0,097	4	0,000	16	0,000
134	0,007	25	0,097	5	0,000	17	0,000
7	0,004	106	0,097	6	0,000	18	0,000
66	0,004	124	0,097	7	0,000	19	0,000
80	0,004	22	0,093	8	0,000	20	0,000
89	0,004	30	0,093	9	0,000	21	0,000
133	0,004	47	0,093	10	0,000	23	0,000
2	0,000	49	0,093	11	0,000	24	0,000
3	0,000	65	0,093	13	0,000	25	0,000
4	0,000	126	0,093	14	0,000	26	0,000
5	0,000	18	0,086	15	0,000	27	0,000
6	0,000	66	0,086	16	0,000	28	0,000
8	0,000	13	0,043	17	0,000	29	0,000
9	0,000	80	0,043	19	0,000	30	0,000
10	0,000	115	0,043	20	0,000	31	0,000
14	0,000	130	0,043	21	0,000	32	0,000

- >50
- >20
- 10-20
- 5-10
- 1-5
- 0.5-1
- 0.1-0.5
- 0.05-0.1
- 0.01-0.05
- 0.005-0.01
- <0.005

APPENDIX -8: Environmental taxa in fish skin wounds.

	C2W3		C2W8		C2W13		C3W4
283	94,4969	227	42,0398	283	99,5747	241	97,6889
71	2,59299	71	18,213	41	0,04455	71	1,44561
190	0,14178	232	2,0988	227	0,03645	283	0,70899
64	0,11939	241	1,50367	158	0,0081	227	0,07366
232	0,07462	64	1,28891	218	0,0081	64	0,0046
70	0,05596	70	1,04776	72	0,00405	178	0,0046
55	0,01119	283	0,70332	113	0,00405	190	0,0046
158	0,01119	62	0,50318	153	0,00405	237	0,0046
222	0,01119	187	0,4766	182	0,00405	1	0
144	0,00746	72	0,23322	243	0,00405	2	0
227	0,00746	8	0,19324	1	0	3	0
45	0,00373	27	0,18999	2	0	4	0
47	0,00373	190	0,17294	3	0	5	0
62	0,00373	45	0,09662	4	0	6	0
159	0,00373	84	0,09662	5	0	7	0
174	0,00373	153	0,09662	6	0	8	0
218	0,00373	182	0,09662	7	0	9	0
224	0,00373	225	0,09662	8	0	10	0
233	0,00373	272	0,09662	9	0	11	0
241	0,00373	55	0,09337	10	0	12	0
243	0,00373	269	0,09337	11	0	13	0
1		158	0,04323	12	0	14	0
2		159	0,04323	13	0	15	0
3		218	0,04323	14	0	16	0
4		233	0,04323	15	0	17	0
5		240	0,04323	16	0	18	0
6		242	0,04323	17	0	19	0

>50
>20
10-20
5-10
1-5
0.5-1
0.1-0.5
0.05-0.1
0.01-0.05
0.005-0.01
<0.005

APPENDIX -9: Inoculums associated taxa in fish skin.

C2W13$_{s.n.w}$		C2W13$_{d.n.w}$		C3W4$_{s.n.w}$		C3W8$_{hf}$		C3W8$_{hf\ gill}$		C4W10$_{lashing}$		C4W10$_{petechia}$		C4W10$_{hf}$	
138	15,272	104	12,530	104	20,880	127	10,441	134	12,627	65	29,504	104	37,575	98	22,944
95	4,422	95	10,362	108	14,498	71	10,232	71	11,895	34	16,471	48	5,944	113	20,584
104	3,559	99	4,699	95	6,475	138	8,892	65	9,192	94	7,962	99	5,057	99	4,573
34	3,387	34	4,170	83	5,735	34	7,290	108	8,671	86	6,409	71	3,957	95	3,162
99	2,071	18	4,098	126	4,053	99	5,657	12	4,476	71	5,931	129	3,558	138	2,899
71	1,186	12	2,659	34	2,754	126	4,808	85	3,941	83	4,213	126	3,106	108	2,525
85	1,143	120	2,295	99	1,511	108	4,126	96	3,252	12	2,666	12	2,209	83	1,220
12	0,992	71	1,930	71	1,299	104	3,726	95	3,069	85	2,585	95	1,972	129	1,194
139	0,733	56	1,493	65	0,689	85	3,660	34	2,829	114	2,582	108	1,912	104	0,856
18	0,690	126	1,421	12	0,462	103	2,320	126	2,675	103	2,397	34	1,350	34	0,770
37	0,539	85	0,874	134	0,319	18	2,284	13	2,660	99	1,951	98	1,296	48	0,617
120	0,431	124	0,656	96	0,305	134	1,997	30	2,013	128	1,896	86	1,221	139	0,536
126	0,410	110	0,619	85	0,263	95	1,800	127	1,365	89	1,825	128	0,756	102	0,506
128	0,324	86	0,492	124	0,226	83	1,734	120	1,140	118	1,553	38	0,738	71	0,482
17	0,259	22	0,437	76	0,217	12	1,728	86	1,126	139	0,835	17	0,531	86	0,298
11	0,237	11	0,419	127	0,199	139	1,645	11	1,056	18	0,796	130	0,395	12	0,295
70	0,173	139	0,346	86	0,148	120	1,639	70	0,929	96	0,764	11	0,354	85	0,243
54	0,108	128	0,328	17	0,143	70	1,208	104	0,774	138	0,508	139	0,246	130	0,234
65	0,108	130	0,291	18	0,125	96	1,053	38	0,732	133	0,443	85	0,228	17	0,232
86	0,108	115	0,273	30	0,120	38	0,909	22	0,577	17	0,404	83	0,186	126	0,208
96	0,108	17	0,255	37	0,102	65	0,658	54	0,535	76	0,382	119	0,181	103	0,200
49	0,086	32	0,237	110	0,088	128	0,640	17	0,521	115	0,356	134	0,175	1	0,191
56	0,086	138	0,200	54	0,083	118	0,628	18	0,479	95	0,343	124	0,171	119	0,137
57	0,086	57	0,182	139	0,083	130	0,610	66	0,479	124	0,265	138	0,149	65	0,080
118	0,086	94	0,146	115	0,074	110	0,490	128	0,465	130	0,223	113	0,145	128	0,063
124	0,086	30	0,127	48	0,069	78	0,431	57	0,408	122	0,210	18	0,119	38	0,059
130	0,086	70	0,127	57	0,051	11	0,377	56	0,310	108	0,142	66	0,103	13	0,054
30	0,065	96	0,127	70	0,051	13	0,299	78	0,282	104	0,110	13	0,073	18	0,046
32	0,065	13	0,109	13	0,042	17	0,281	99	0,239	13	0,087	54	0,055	89	0,046
83	0,065	54	0,091	56	0,042	57	0,233	55	0,211	134	0,087	57	0,045	66	0,044
16	0,043	111	0,091	11	0,037	115	0,215	83	0,141	135	0,049	120	0,041	124	0,041
22	0,043	133	0,091	22	0,032	76	0,209	109	0,113	70	0,036	30	0,030	112	0,037
91	0,043	66	0,073	6	0,028	135	0,197	80	0,099	110	0,036	102	0,026	54	0,033
110	0,043	129	0,073	1	0,023	54	0,173	111	0,084	120	0,036	70	0,019	70	0,022
111	0,043	29	0,055	38	0,023	30	0,167	6	0,070	11	0,032	133	0,015	57	0,019
131	0,043	76	0,055	120	0,023	56	0,167	16	0,070	37	0,023	111	0,013	30	0,013
1	0,022	78	0,055	128	0,023	86	0,155	19	0,070	80	0,019	55	0,011	134	0,013
15	0,022	127	0,055	55	0,018	37	0,150	42	0,070	126	0,019	96	0,009	76	0,011
38	0,022	16	0,036	67	0,018	124	0,144	61	0,070	1	0,016	103	0,009	11	0,009
61	0,022	27	0,036	89	0,018	89	0,138	130	0,070	38	0,013	112	0,009	115	0,007
103	0,022	55	0,036	111	0,018	114	0,138	115	0,056	57	0,013	115	0,008	133	0,007
129	0,022	106	0,036	16	0,014	109	0,120	124	0,056	2	0,010	65	0,006	94	0,006
134	0,022	108	0,036	61	0,014	132	0,114	133	0,056	54	0,010	89	0,004	55	0,004
135	0,022	131	0,036	66	0,009	111	0,102	41	0,042	78	0,010	56	0,002	22	0,000
2	0,000	1	0,018	118	0,009	19	0,078	132	0,042	106	0,010	76	0,002	56	0,000
3	0,000	5	0,018	129	0,009	66	0,048	36	0,028	111	0,010	1	0,000	96	0,000
4	0,000	15	0,018	133	0,009	6	0,042	48	0,028	112	0,010	22	0,000	111	0,000
5	0,000	38	0,018	2	0,005	131	0,030	67	0,028	116	0,010	94	0,000	120	0,000
6	0,000	61	0,018	27	0,005	133	0,030	89	0,028	129	0,010	127	0,000	127	0,000
7	0,000	65	0,018	32	0,005	22	0,024	110	0,028	132	0,010	2		2	
8	0,000	67	0,018	46	0,005	55	0,024	125	0,028	19	0,006	3		3	
9	0,000	80	0,018	49	0,005	94	0,018	138	0,028	91	0,006	4		4	
10	0,000	83	0,018	80	0,005	9	0,012	139	0,028	10	0,003	5		5	
13	0,000	103	0,018	94	0,005	16	0,012	1	0,014	16	0,003	6		6	
14	0,000	109	0,018	116	0,005	42	0,012	2	0,014	22	0,003	7		7	
19	0,000	135	0,018	135	0,005	1	0,006	29	0,014	25	0,003	8		8	
20	0,000	2	0,000	136	0,005	2	0,006	32	0,014	46	0,003	9		9	
21	0,000	3	0,000	3	0,000	5	0,006	46	0,014	53	0,003	10		10	
23	0,000	4	0,000	4	0,000	32	0,006	106	0,014	56	0,003	14		14	
24	0,000	6	0,000	5	0,000	49	0,006	129	0,014	64	0,003	15		15	
25	0,000	7	0,000	7	0,000	61	0,006	131	0,014	109	0,003	16		16	
26	0,000	8	0,000	8	0,000	80	0,006	3	0,000	119	0,003	19		19	
27	0,000	9	0,000	9	0,000	121	0,006	4	0,000	3	0,000	20		20	
28	0,000	10	0,000	10	0,000	122	0,006	5	0,000	4	0,000	21		21	
29	0,000	14	0,000	14	0,000	129	0,006	7	0,000	5	0,000	23		23	

>50
>20
10-20
5-10
1-5
0.5-1
0.1-0.5
0.05-0.1
0.01-0.05
0.005-0.01
<0.005

APPENDIX -10: Environmental taxa in fish skin.

C2W13$_{s.n.w}$		C2W13$_{d.n.w}$		C3W4$_{s.n.w}$		C3W8$_{hf}$		C3W8$_{hf\ gill}$		C4W10$_{lashing}$		C4W10$_{petechia}$		C4W10$_{hf}$	
243	58,305	283	41,668	241	20,422	64	2,978	243	4,251	190	1,178	70	2,890	283	0,823
64	1,553	150	0,765	283	9,922	283	1,836	283	3,252	64	0,802	63	2,043	70	0,513
227	0,539	64	0,692	227	4,561	243	1,674	64	1,886	240	0,615	158	1,432	72	0,495
72	0,453	227	0,692	233	0,615	227	1,621	227	1,450	66	0,573	237	0,760	158	0,417
269	0,302	55	0,619	64	0,582	66	1,041	70	0,901	158	0,466	152	0,452	63	0,397
70	0,173	187	0,437	224	0,564	267	0,825	218	0,859	243	0,434	69	0,433	166	0,345
283	0,173	153	0,419	38	0,236	229	0,664	241	0,802	159	0,262	55	0,331	71	0,323
41	0,151	72	0,255	150	0,203	242	0,628	187	0,760	237	0,207	248	0,305	243	0,263
242	0,108	224	0,200	243	0,166	54	0,431	150	0,563	271	0,165	164	0,188	65	0,124
55	0,086	51	0,146	27	0,129	222	0,359	190	0,479	29	0,139	267	0,175	237	0,117
98	0,086	124	0,146	232	0,116	70	0,293	229	0,380	153	0,120	153	0,166	152	0,106
150	0,065	159	0,109	153	0,111	224	0,293	124	0,338	70	0,110	71	0,090	55	0,093
241	0,065	29	0,091	190	0,106	150	0,263	237	0,296	55	0,065	166	0,053	69	0,056
66	0,043	158	0,073	230	0,092	190	0,257	267	0,253	204	0,065	72	0,043	267	0,030
71	0,043	218	0,073	158	0,083	80	0,245	159	0,239	189	0,049	154	0,036	154	0,028
135	0,043	71	0,055	187	0,079	57	0,233	224	0,225	224	0,036	27	0,026	241	0,019
138	0,043	222	0,055	159	0,055	233	0,191	182	0,211	236	0,036	150	0,024	164	0,017
153	0,043	42	0,036	62	0,051	55	0,138	158	0,197	261	0,029	243	0,023	153	0,013
232	0,043	45	0,036	70	0,037	187	0,132	50	0,183	306	0,026	283	0,017	27	0,011
236	0,043	50	0,036	44	0,032	111	0,114	233	0,183	61	0,023	65	0,008	168	0,007
256	0,043	75	0,036	71	0,032	72	0,102	111	0,127	194	0,023	241	0,006	156	0,006
29	0,022	123	0,036	123	0,032	159	0,102	153	0,127	27	0,019	168	0,004	150	0,004
30	0,022	190	0,036	237	0,032	153	0,084	232	0,113	139	0,019	124	0,000	248	0,004
40	0,022	233	0,036	138	0,028	218	0,084	19	0,099	193	0,016	155	0,000	124	0,000
42	0,022	40	0,018	236	0,018	27	0,078	27	0,099	310	0,016	156	0,000	155	0,000
75	0,022	41	0,018	21	0,014	158	0,072	55	0,099	19	0,013	161	0,000	161	0,000
157	0,022	65	0,018	54	0,014	135	0,066	72	0,070	78	0,013	233	0,000	233	0,000
158	0,022	66	0,018	72	0,014	50	0,054	113	0,070	230	0,013	240	0,000	240	0,000
229	0,022	82	0,018	124	0,014	241	0,054	178	0,070	21	0,010	1	0,000	1	0,000
278	0,022	84	0,018	222	0,014	44	0,042	204	0,070	45	0,010	2	0,000	2	0,000
1	0,000	135	0,018	242	0,014	230	0,042	282	0,070	74	0,010	3	0,000	3	0,000
2	0,000	138	0,018	19	0,009	41	0,036	21	0,056	178	0,010	4	0,000	4	0,000
3	0,000	141	0,018	85	0,009	78	0,036	41	0,056	242	0,010	5	0,000	5	0,000
4	0,000	191	0,018	102	0,009	237	0,036	45	0,056	50	0,006	6	0,000	6	0,000
5	0,000	192	0,018	135	0,009	240	0,036	222	0,056	54	0,006	7	0,000	7	0,000
6	0,000	236	0,018	204	0,009	270	0,036	18	0,042	93	0,006	8	0,000	8	0,000
7	0,000	241	0,018	215	0,009	94	0,030	44	0,042	145	0,006	9	0,000	9	0,000
8	0,000	269	0,018	218	0,009	124	0,030	51	0,042	164	0,006	10	0,000	10	0,000
9	0,000	276	0,018	240	0,009	204	0,030	167	0,042	192	0,006	11	0,000	11	0,000
10	0,000	1	0,000	20	0,005	310	0,030	54	0,028	235	0,006	12	0,000	12	0,000
11	0,000	2	0,000	78	0,005	108	0,024	114	0,028	307	0,006	13	0,000	13	0,000
12	0,000	3	0,000	141	0,005	178	0,024	135	0,028	20	0,003	14	0,000	14	0,000
13	0,000	4	0,000	154	0,005	304	0,024	154	0,028	72	0,003	15	0,000	15	0,000
14	0,000	5	0,000	167	0,005	19	0,018	236	0,028	85	0,003	16	0,000	16	0,000
15	0,000	6	0,000	178	0,005	29	0,018	259	0,028	149	0,003	17	0,000	17	0,000
16	0,000	7	0,000	182	0,005	71	0,018	295	0,028	171	0,003	18	0,000	18	0,000
17	0,000	8	0,000	229	0,005	228	0,018	29	0,014	175	0,003	19	0,000	19	0,000
18	0,000	9	0,000	244	0,005	266	0,018	42	0,014	187	0,003	20	0,000	20	0,000
19	0,000	10	0,000	265	0,005	100	0,012	85	0,014	222	0,003	21	0,000	21	0,000
20	0,000	11	0,000	269	0,005	116	0,012	94	0,014	227	0,003	22	0,000	22	0,000
21	0,000	12	0,000	306	0,005	139	0,012	101	0,014	232	0,003	23	0,000	23	0,000
22	0,000	13	0,000	1	0,000	236	0,012	116	0,014	238	0,003	24	0,000	24	0,000
23	0,000	14	0,000	2	0,000	244	0,012	119	0,014	241	0,003	25	0,000	25	0,000
24	0,000	15	0,000	3	0,000	261	0,012	123	0,014	248	0,003	26	0,000	26	0,000
25	0,000	16	0,000	4	0,000	2	0,006	138	0,014	262	0,003	28	0,000	28	0,000
26	0,000	17	0,000	5	0,000	18	0,006	145	0,014	1	0,000	29	0,000	29	0,000
27	0,000	18	0,000	6	0,000	51	0,006	156	0,014	2	0,000	30	0,000	30	0,000
28	0,000	19	0,000	7	0,000	58	0,006	175	0,014	3	0,000	31	0,000	31	0,000
31	0,000	20	0,000	8	0,000	96	0,006	188	0,014	4	0,000	32	0,000	32	0,000
32	0,000	21	0,000	9	0,000	138	0,006	193	0,014	5	0,000	33	0,000	33	0,000
33	0,000	22	0,000	10	0,000	141	0,006	228	0,014	6	0,000	34	0,000	34	0,000
34	0,000	23	0,000	11	0,000	154	0,006	230	0,014	7	0,000	35	0,000	35	0,000
35	0,000	24	0,000	12	0,000	167	0,006	235	0,014	8	0,000	36	0,000	36	0,000
36	0,000	25	0,000	13	0,000	174	0,006	240	0,014	9	0,000	37	0,000	37	0,000
37	0,000	26	0,000	14	0,000	194	0,006	245	0,014	10	0,000	38	0,000	38	0,000
38	0,000	27	0,000	15	0,000	225	0,006	281	0,014	11	0,000	39	0,000	39	0,000
39	0,000	28	0,000	16	0,000	232	0,006	296	0,014	12	0,000	40	0,000	40	0,000
43	0,000	30	0,000	17	0,000	253	0,006	298	0,014	13	0,000	41	0,000	41	0,000
44	0,000	31	0,000	18	0,000	256	0,006	1	0,000	14	0,000	42	0,000	42	0,000
45	0,000	32	0,000	22	0,000	265	0,006	2	0,000	15	0,000	43	0,000	43	0,000
46	0,000	33	0,000	23	0,000	295	0,006	3	0,000	16	0,000	44	0,000	44	0,000

>50
>20
10-20
5-10
1-5
0.5-1
0.1-0.5
0.05-0.1
0.01-0.05
0.005-0.01
<0.005

APPENDIX-11: Number code for inoculums associated taxa

1	Archaea;__Thaumarchaeota;__Soil_Crenarchaeotic_Group(SCG);__o;__f;__g
2	Bacteria;__Proteobacteria;__Alphaproteobacteria;__Rhizobiales;__Bradyrhizobiaceae;__Bradyrhizobium
3	Bacteria;__Cyanobacteria;__MLE1-12;__o;__f;__g
4	Archaea;__Thaumarchaeota;__Sc-EA05;__o;__f;__g
5	Bacteria;__Acidobacteria;__Acidobacteria;__Candidatus_Chloracidobacterium;__f;__g
6	Bacteria;__TM6;__c;__o;__f;__g
7	Bacteria;__Actinobacteria;__Acidimicrobiia;__Acidimicrobiales;__OCS155_marine_group;__g
8	Bacteria;__Deinococcus-Thermus;__Deinococci;__Deinococcales;__Trueperaceae;__Truepera
9	Bacteria;__Bacteroidetes;__Flavobacteria;__Flavobacteriales;__Flavobacteriaceae;__Arenibacter
10	Bacteria;__Proteobacteria;__Alphaproteobacteria;__Rhizobiales;__Xanthobacteraceae;__Pseudolabrys
11	Bacteria;__Bacteroidetes;__Sphingobacteriia;__Sphingobacteriales;__Saprospiraceae;__Lewinella
12	Unassigned;Other;Other;Other;Other;Other
13	Bacteria;__Planctomycetes;__Phycisphaerae;__Phycisphaerales;__Phycisphaeraceae;__Phycisphaera
14	Bacteria;__Actinobacteria;__Acidimicrobiia;__Acidimicrobiales;__TM214;__g
15	Bacteria;__Proteobacteria;__Gammaproteobacteria;__NKB5;__f;__g
16	Bacteria;__Proteobacteria;__Deltaproteobacteria;__Sh765B-TzT-29;__f;__g
17	Bacteria;__Proteobacteria;__Gammaproteobacteria;__Order_Incertae_Sedis;__Family_Incertae_Sedis;__Marinicella
18	Bacteria;__Bacteroidetes;__Sphingobacteriia;__Sphingobacteriales;__Chitinophagaceae;__g
19	Bacteria;__Bacteroidetes;__Sphingobacteriia;__Sphingobacteriales;__NS11-12_marine_group;__g
20	Bacteria;__Proteobacteria;__Alphaproteobacteria;__Caulobacterales;__Hyphomonadaceae;__Woodsholea
21	Bacteria;__Chlorobi;__Ignavibacteria;__Ignavibacteriales;__PHOS-HE36;__g
22	Bacteria;__Chloroflexi;__Anaerolineae;__Anaerolineales;__Anaerolineaceae;__g
23	Bacteria;__Chloroflexi;__TK10;__o;__f;__g
24	Bacteria;__Proteobacteria;__Gammaproteobacteria;__Pseudomonadales;__Pseudomonadaceae;__Cellvibrio
25	Bacteria;__Chloroflexi;__Caldilineae;__Caldilineales;__Caldilineaceae;__Caldilinea
26	Bacteria;__Gemmatimonadetes;__Gemmatimonadetes;__Gemmatimonadales;__Gemmatimonadaceae;__g
27	Bacteria;__Verrucomicrobia;__Opitutae;__Opitutales;__Opitutaceae;__Opitutus
28	Bacteria;__Proteobacteria;__Betaproteobacteria;__Nitrosomonadales;__Nitrosomonadaceae;__g
29	Bacteria;__Planctomycetes;__Planctomycetacia;__Planctomycetales;__Planctomycetaceae;__g
30	Bacteria;__Planctomycetes;__OM190;__o;__f;__g
31	Bacteria;__Planctomycetes;__Phycisphaerae;__Phycisphaerales;__Phycisphaeraceae;Other
32	Bacteria;__Verrucomicrobia;__OPB35_soil_group;__o;__f;__g
33	Bacteria;__Candidate_division_WS3;__c;__o;__f;__g
34	Bacteria;__Proteobacteria;__Alphaproteobacteria;__Rhodobacterales;__Rhodobacteraceae;__g
35	Bacteria;__Chloroflexi;__Gitt-GS-136;__o;__f;__g
36	Bacteria;__Actinobacteria;__Acidimicrobiia;__Acidimicrobiales;__Iamiaceae;__Iamia
37	Bacteria;__Proteobacteria;__Gammaproteobacteria;__Pseudomonadales;__Pseudomonadaceae;__Pseudomonas
38	Bacteria;__Bacteroidetes;__Sphingobacteriia;__Sphingobacteriales;__Saprospiraceae;__g
39	Bacteria;__Proteobacteria;__Gammaproteobacteria;__Xanthomonadales;__Sinobacteraceae;__g
40	Bacteria;__Chloroflexi;__Thermomicrobia;__JG30-KF-CM45;__f;__g
41	Bacteria;__Bacteroidetes;__Cytophagia;__Order_II_Incertae_Sedis;__Rhodothermaceae;__g
42	Bacteria;__Proteobacteria;__Deltaproteobacteria;__Myxococcales;__Sorangiineae;__g
43	Bacteria;__Armatimonadetes;__c;__o;__f;__g
44	Bacteria;__Proteobacteria;__Gammaproteobacteria;__Legionellales;__Coxiellaceae;__Aquicella
45	Bacteria;__Bacteroidetes;__Sphingobacteriia;__Sphingobacteriales;__env.OPS_17;__g
46	Bacteria;__Proteobacteria;__Deltaproteobacteria;__Myxococcales;__0319-6G20;__g
47	Bacteria;__Proteobacteria;Other;Other;Other;Other
48	Bacteria;__Proteobacteria;__Deltaproteobacteria;__Myxococcales;__Nannocystaceae;__Nannocystis
49	Bacteria;__Proteobacteria;__Betaproteobacteria;__Burkholderiales;__Comamonadaceae;__Pelomonas
50	Bacteria;__Chloroflexi;__Thermomicrobia;__AKYG1722;__f;__g
51	Bacteria;__Chloroflexi;__KD4-96;__o;__f;__g
52	Bacteria;__Proteobacteria;__Gammaproteobacteria;__Xanthomonadales;__Xanthomonadaceae;__Pseudofulvimonas
53	Bacteria;__Proteobacteria;__Gammaproteobacteria;__aaa34a10;__f;__g
54	Bacteria;__Planctomycetes;__Planctomycetacia;__Planctomycetales;__Planctomycetaceae;__Pir4_lineage
55	Bacteria;__Proteobacteria;__Deltaproteobacteria;__Myxococcales;__Sandaracinaceae;__Sandaracinus
56	Bacteria;__Proteobacteria;__Alphaproteobacteria;__Rhodobacterales;__Rhodobacteraceae;__Pseudorhodobacter
57	Bacteria;__Planctomycetes;__Planctomycetacia;__Planctomycetales;__Planctomycetaceae;__Planctomyces
58	Bacteria;__Candidate_division_TM7;__c;__o;__f;__g
59	Bacteria;__Planctomycetes;__Planctomycetacia;__Planctomycetales;__Planctomycetaceae;__Gemmata
60	Bacteria;__Chloroflexi;__Caldilineae;__Caldilineales;__Caldilineaceae;__g
61	Bacteria;__Proteobacteria;__Alphaproteobacteria;__Rhodospirillales;__Rhodospirillaceae;__g
62	Bacteria;__Proteobacteria;__Alphaproteobacteria;__Rickettsiales;__Rickettsiaceae;__g
63	Bacteria;__Proteobacteria;__Betaproteobacteria;__Burkholderiales;__Comamonadaceae;Other
64	Bacteria;__Proteobacteria;__Deltaproteobacteria;__Bdellovibrionales;__Bacteriovoracaceae;__Peredibacter
65	Bacteria;__Proteobacteria;__Deltaproteobacteria;__Desulfuromonadales;__GR-WP33-58;__g
66	Bacteria;__Proteobacteria;__Betaproteobacteria;__Nitrosomonadales;__Nitrosomonadaceae;__Nitrosomonas
67	Bacteria;__Verrucomicrobia;__Verrucomicrobiae;__Verrucomicrobiales;__DEV007;__g
68	Bacteria;__Actinobacteria;__Acidimicrobiia;__Acidimicrobiales;__uncultured;__g
69	Bacteria;__Acidobacteria;__Acidobacteria;__DA023;__f;__g
70	Bacteria;__Proteobacteria;__Alphaproteobacteria;__Rhodobacterales;__Rhodobacteraceae;__Rhodobacter
71	Bacteria;__Proteobacteria;__Alphaproteobacteria;__Rhodobacterales;__Rhodobacteraceae;Other
72	Bacteria;__Proteobacteria;__Alphaproteobacteria;__Rhodospirillales;__Rhodospirillaceae;__Rhodovibrio
73	Bacteria;__Chloroflexi;__S085;__o;__f;__g
74	Bacteria;__Elusimicrobia;__Elusimicrobia;__Lineage_IIb;__f;__g
75	Bacteria;__Proteobacteria;__Alphaproteobacteria;__Rhodospirillales;__wr0007;__g
76	Bacteria;__Chlorobi;__Chlorobia;__Chlorobiales;__OPB56;__g
77	Bacteria;__Proteobacteria;__Alphaproteobacteria;__Rickettsiales;__EF100-94H03;__g
78	Bacteria;__Cyanobacteria;__Chloroplast;__Chloroplast;__Chloroplast;__Chloroplast
79	Bacteria;__Proteobacteria;__Alphaproteobacteria;__Rhizobiales;__Hyphomicrobiaceae;__Hyphomicrobium
80	Bacteria;__Planctomycetes;__Phycisphaerae;__Phycisphaerales;__Phycisphaeraceae;__SM1A02
81	Bacteria;__Proteobacteria;__Gammaproteobacteria;__Order_Incertae_Sedis;__Family_Incertae_Sedis;__Thiohalophilus
82	Bacteria;__Chloroflexi;__JG30-KF-CM66;__o;__f;__g
83	Bacteria;__Proteobacteria;__Gammaproteobacteria;__Alteromonadales;__Alteromonadaceae;__Marinobacter
84	Bacteria;__Planctomycetes;__Planctomycetacia;__Planctomycetales;__Planctomycetaceae;__Singulisphaera
85	Bacteria;__Bacteroidetes;__Flavobacteria;__Flavobacteriales;__Cryomorphaceae;__Owenweeksia
86	Bacteria;__Bacteroidetes;__Cytophagia;__Cytophagales;__Cytophagaceae;__Flexibacter
87	Bacteria;__Bacteroidetes;__Sphingobacteriia;__Sphingobacteriales;__Sphingobacteriaceae;__Pedobacter
88	Bacteria;__Proteobacteria;__Betaproteobacteria;__TRA3-20;__f;__g
89	Bacteria;__Proteobacteria;__Gammaproteobacteria;__Oceanospirillales;__Alcanivoracaceae;__Alcanivorax
90	Bacteria;__Actinobacteria;__Thermoleophilia;__Solirubrobacterales;__480-2;__g
91	Bacteria;__Proteobacteria;__Alphaproteobacteria;__Sphingomonadales;__Erythrobacteraceae;__Erythrobacter
92	Bacteria;__Proteobacteria;__Alphaproteobacteria;__Rickettsiales;__f;__g
93	Bacteria;__WCHB1-60;__c;__o;__f;__g
94	Bacteria;__Bacteroidetes;__Flavobacteria;__Flavobacteriales;__Flavobacteriaceae;__Aequorivita
95	Bacteria;__Proteobacteria;__Gammaproteobacteria;__Alteromonadales;__Colwelliaceae;__Colwellia
96	Bacteria;__Proteobacteria;__Gammaproteobacteria;__Alteromonadales;__Alteromonadaceae;__BD1-7_clade
97	Bacteria;__Proteobacteria;__SC3-20;__o;__f;__g
98	Bacteria;__Firmicutes;__Bacilli;__Bacillales;__Thermoactinomycetaceae;Other
99	Bacteria;__Bacteroidetes;__Flavobacteria;__Flavobacteriales;__Flavobacteriaceae;Other
100	Bacteria;__Proteobacteria;__Deltaproteobacteria;__Syntrophobacterales;__Syntrophaceae;__Smithella

101	Bacteria;__Proteobacteria;__Gammaproteobacteria;__Legionellales;__Legionellaceae;__Legionella
102	Bacteria;__Chlorobi;__Chlorobia;__Chlorobiales;__SJA-28;__g
103	Bacteria;__Bacteroidetes;__Flavobacteria;__Flavobacteriales;__Cryomorphaceae;__Brumimicrobium
104	Bacteria;__Bacteroidetes;__Sphingobacteriia;__Sphingobacteriales;__Saprospiraceae;__Aureispira
105	Bacteria;__Proteobacteria;__Alphaproteobacteria;__Rhizobiales;__uncultured;__g
106	Bacteria;__Nitrospirae;__Nitrospira;__Nitrospirales;__Nitrospiraceae;__Nitrospira
107	Bacteria;__Proteobacteria;__Alphaproteobacteria;__Rhodospirillales;__Rhodospirillaceae;Other
108	Bacteria;__Proteobacteria;__Gammaproteobacteria;__Alteromonadales;__Alteromonadaceae;__Glaciecola
109	Bacteria;__Proteobacteria;__Gammaproteobacteria;__Thiotrichales;__Francisellaceae;__Francisella
110	Bacteria;__Proteobacteria;__Alphaproteobacteria;__Rhizobiales;__Phyllobacteriaceae;__Ahrensia
111	Bacteria;__Planctomycetes;__Planctomycetacia;__Planctomycetales;__Planctomycetaceae;__Rhodopirellula
112	Bacteria;__Proteobacteria;__Alphaproteobacteria;__Rhizobiales;__Rhodobiaceae;__Parvibaculum
113	Bacteria;__Acidobacteria;__Holophagae;__NS72;__f;__g
114	Bacteria;__Bacteroidetes;__Cytophagia;__Order_III_Incertae_Sedis;__Family_Incertae_Sedis;__Balneola
115	Bacteria;__Proteobacteria;__Alphaproteobacteria;__Rhodobacterales;__Rhodobacteraceae;__Paracoccus
116	Bacteria;__Proteobacteria;__Gammaproteobacteria;__Xanthomonadales;__Sinobacteraceae;__JTB255_marine_benthic_group
117	Bacteria;__Proteobacteria;__Alphaproteobacteria;__Rhizobiales;__Phyllobacteriaceae;Other
118	Bacteria;__Proteobacteria;__Gammaproteobacteria;__Oceanospirillales;__MBAE14;__g
119	Bacteria;__Bacteroidetes;__Flavobacteria;__Flavobacteriales;__Flavobacteriaceae;__Muricauda
120	Bacteria;__Verrucomicrobia;__Verrucomicrobiae;__Verrucomicrobiales;__Verrucomicrobiaceae;__Persicirhabdus
121	Bacteria;__Actinobacteria;__Actinobacteria;__Micrococcales;__Micrococcaceae;__Arthrobacter
122	Bacteria;__Bacteroidetes;__Cytophagia;__Cytophagales;__Cyclobacteriaceae;__Cyclobacterium
123	Bacteria;__Fibrobacteres;__Fibrobacteria;__Fibrobacterales;__Fibrobacteraceae;__g
124	Bacteria;__Proteobacteria;__Alphaproteobacteria;__DB1-14;__f;__g
125	Bacteria;__Proteobacteria;__Alphaproteobacteria;__Rickettsiales;__Candidatus_Odyssella;__g
126	Bacteria;__Bacteroidetes;__Cytophagia;__Cytophagales;__Cytophagaceae;__Leadbetterella
127	Bacteria;__Bacteroidetes;__Flavobacteria;__Flavobacteriales;__Cryomorphaceae;__Lishizhenia
128	Bacteria;__Bacteroidetes;__Flavobacteria;__Flavobacteriales;__Flavobacteriaceae;__Lutibacter
129	Bacteria;__Bacteroidetes;__Flavobacteria;__Flavobacteriales;__Flavobacteriaceae;__g
130	Bacteria;__Bacteroidetes;__Flavobacteria;__Flavobacteriales;__NS9_marine_group;__g
131	Bacteria;__Planctomycetes;__Planctomycetacia;__Planctomycetales;__Planctomycetaceae;__Pirellula
132	Bacteria;__Proteobacteria;__Alphaproteobacteria;__Rickettsiales;__mitochondria;__g
133	Bacteria;__Proteobacteria;__Alphaproteobacteria;__Sneathiellales;__Sneathiellaceae;__Sneathiella
134	Bacteria;__Proteobacteria;__Deltaproteobacteria;__Myxococcales;__Nannocystineae;__Nannocystaceae
135	Bacteria;__Proteobacteria;__Gammaproteobacteria;__Alteromonadales;__Idiomarinaceae;__Idiomarina
136	Bacteria;__Proteobacteria;__Gammaproteobacteria;__Oceanospirillales;__Alcanivoracaceae;__Kangiella
137	Bacteria;__Proteobacteria;__Gammaproteobacteria;__Order_Incertae_Sedis;__Family_Incertae_Sedis;Other
138	Bacteria;__Proteobacteria;__Gammaproteobacteria;__Pseudomonadales;__Moraxellaceae;__Psychrobacter
139	Bacteria;__Verrucomicrobia;__Verrucomicrobiae;__Verrucomicrobiales;__Rubritaleaceae;__Rubritalea

APPENDIX-12: Number code for environmental taxa.

1	Archaea;__Euryarchaeota;__Halobacteria;__Halobacteriales;__Deep_Sea_Hydrothermal_Vent_Gp_6(DHVEG-6);__Candidatus_Parvarchaeum
2	Archaea;__Euryarchaeota;__Halobacteria;__Halobacteriales;__Deep_Sea_Hydrothermal_Vent_Gp_6(DHVEG-6);__g
3	Archaea;__Euryarchaeota;__Halobacteria;__Halobacteriales;__Halobacteriaceae;__g
4	Archaea;__Euryarchaeota;__Methanococci;__Methanococcales;__Methanococcaceae;__Methanococcus
5	Archaea;__Euryarchaeota;__Thermoplasmata;__Thermoplasmatales;__Marine_Group_II;__g
6	Archaea;__Euryarchaeota;__Thermoplasmata;__Thermoplasmatales;__Marine_Group_III;__g
7	Archaea;__Thaumarchaeota;__Marine_Benthic_Group_A;__o;__f;__g
8	Archaea;__Thaumarchaeota;__Marine_Group_I;__o;__f;__g
9	Bacteria;Other;Other;Other;Other;Other
10	Bacteria;__Acidobacteria;__Acidobacteria;__PAUC26f;__f;__g
11	Bacteria;__Acidobacteria;__Holophagae;__Acanthopleuribacterales;__Acanthopleuribacteraceae;__Acanthopleuribacter
12	Bacteria;__Acidobacteria;__Holophagae;__CA002;__f;__g
13	Bacteria;__Acidobacteria;__Holophagae;__NKB17;__f;__g
14	Bacteria;__Acidobacteria;__Holophagae;__Sva0725;__f;__g
15	Bacteria;__Acidobacteria;__RB25;__o;__f;__g
16	Bacteria;__Actinobacteria;__Acidimicrobiia;__Acidimicrobiales;Other;Other
17	Bacteria;__Actinobacteria;__Acidimicrobiia;__Acidimicrobiales;__Acidimicrobiaceae;Other
18	Bacteria;__Actinobacteria;__Acidimicrobiia;__Acidimicrobiales;__Candidatus_Microthrix;__g
19	Bacteria;__Actinobacteria;__Acidimicrobiia;__Acidimicrobiales;__Sva0996_marine_group;__g
20	Bacteria;__Actinobacteria;__Actinobacteria;__Corynebacteriales;__Corynebacteriaceae;__Corynebacterium
21	Bacteria;__Actinobacteria;__Actinobacteria;__Corynebacteriales;__Mycobacteriaceae;__Mycobacterium
22	Bacteria;__Actinobacteria;__Actinobacteria;__Micrococcales;__Micrococcaceae;__Micrococcus
23	Bacteria;__Actinobacteria;__Actinobacteria;__Micromonosporales;__Micromonosporaceae;Other
24	Bacteria;__Actinobacteria;__Actinobacteria;__PeM15;__f;__g
25	Bacteria;__Actinobacteria;__Thermoleophilia;__Solirubrobacterales;__Elev-16S-1332;__g
26	Bacteria;__Actinobacteria;__Thermoleophilia;__Solirubrobacterales;__TM146;__g
27	Bacteria;__BD1-5;__c;__o;__f;__g
28	Bacteria;__BHI80-139;__c;__o;__f;__g
29	Bacteria;__Bacteroidetes;Other;Other;Other;Other
30	Bacteria;__Bacteroidetes;__AMV16;__o;__f;__g
31	Bacteria;__Bacteroidetes;__BD2-2;__o;__f;__g
32	Bacteria;__Bacteroidetes;__Bacteroidia;__Bacteroidales;__Bacteroidaceae;__Bacteroides
33	Bacteria;__Bacteroidetes;__Bacteroidia;__Bacteroidales;__Marinilabiaceae;__g
34	Bacteria;__Bacteroidetes;__Bacteroidia;__Bacteroidales;__Porphyromonadaceae;__Paludibacter
35	Bacteria;__Bacteroidetes;__Bacteroidia;__Bacteroidales;__Porphyromonadaceae;__g
36	Bacteria;__Bacteroidetes;__Bacteroidia;__Bacteroidales;__Rikenellaceae;__RC9_gut_group
37	Bacteria;__Bacteroidetes;__Class_Incertae_Sedis;__Order_Incertae_Sedis;__Family_Incertae_Sedis;__Prolixibacter
38	Bacteria;__Bacteroidetes;__Cytophagia;__Cytophagales;Other;Other
39	Bacteria;__Bacteroidetes;__Cytophagia;__Cytophagales;__Cytophagaceae;__Adhaeribacter
40	Bacteria;__Bacteroidetes;__Cytophagia;__Cytophagales;__Cytophagaceae;__Cytophaga
41	Bacteria;__Bacteroidetes;__Cytophagia;__Cytophagales;__Cytophagaceae;__Microscilla
42	Bacteria;__Bacteroidetes;__Cytophagia;__Cytophagales;__Flammeovirgaceae;Other
43	Bacteria;__Bacteroidetes;__Cytophagia;__Cytophagales;__Flammeovirgaceae;__Candidatus_Amoebophilus
44	Bacteria;__Bacteroidetes;__Cytophagia;__Cytophagales;__Flammeovirgaceae;__Ekhidna
45	Bacteria;__Bacteroidetes;__Cytophagia;__Cytophagales;__Flammeovirgaceae;__Flexithrix
46	Bacteria;__Bacteroidetes;__Cytophagia;__Cytophagales;__Flammeovirgaceae;__Marinoscillum
47	Bacteria;__Bacteroidetes;__Cytophagia;__Cytophagales;__Flammeovirgaceae;__Marivirga
48	Bacteria;__Bacteroidetes;__Cytophagia;__Cytophagales;__Flammeovirgaceae;__Persicobacter
49	Bacteria;__Bacteroidetes;__Cytophagia;__Cytophagales;__Flammeovirgaceae;__Rapidithrix
50	Bacteria;__Bacteroidetes;__Cytophagia;__Cytophagales;__Flammeovirgaceae;__Reichenbachiella
51	Bacteria;__Bacteroidetes;__Cytophagia;__Cytophagales;__Flammeovirgaceae;__g
52	Bacteria;__Bacteroidetes;__Flavobacteria;__Flavobacteriales;Other;Other
53	Bacteria;__Bacteroidetes;__Flavobacteria;__Flavobacteriales;__Cryomorphaceae;Other
54	Bacteria;__Bacteroidetes;__Flavobacteria;__Flavobacteriales;__Cryomorphaceae;__Crocinitomix
55	Bacteria;__Bacteroidetes;__Flavobacteria;__Flavobacteriales;__Cryomorphaceae;__Fluviicola
56	Bacteria;__Bacteroidetes;__Flavobacteria;__Flavobacteriales;__Cryomorphaceae;__NS10_marine_group
57	Bacteria;__Bacteroidetes;__Flavobacteria;__Flavobacteriales;__Cryomorphaceae;__NS7_marine_group
58	Bacteria;__Bacteroidetes;__Flavobacteria;__Flavobacteriales;__Flavobacteriaceae;__Cellulophaga
59	Bacteria;__Bacteroidetes;__Flavobacteria;__Flavobacteriales;__Flavobacteriaceae;__Croceibacter
60	Bacteria;__Bacteroidetes;__Flavobacteria;__Flavobacteriales;__Flavobacteriaceae;__Flavobacterium
61	Bacteria;__Bacteroidetes;__Flavobacteria;__Flavobacteriales;__Flavobacteriaceae;__Gramella
62	Bacteria;__Bacteroidetes;__Flavobacteria;__Flavobacteriales;__Flavobacteriaceae;__Kordia
63	Bacteria;__Bacteroidetes;__Flavobacteria;__Flavobacteriales;__Flavobacteriaceae;__Leeuwenhoekiella
64	Bacteria;__Bacteroidetes;__Flavobacteria;__Flavobacteriales;__Flavobacteriaceae;__Maribacter
65	Bacteria;__Bacteroidetes;__Flavobacteria;__Flavobacteriales;__Flavobacteriaceae;__Maritimimonas
66	Bacteria;__Bacteroidetes;__Flavobacteria;__Flavobacteriales;__Flavobacteriaceae;__Mesonia
67	Bacteria;__Bacteroidetes;__Flavobacteria;__Flavobacteriales;__Flavobacteriaceae;__NS4_marine_group
68	Bacteria;__Bacteroidetes;__Flavobacteria;__Flavobacteriales;__Flavobacteriaceae;__NS5_marine_group
69	Bacteria;__Bacteroidetes;__Flavobacteria;__Flavobacteriales;__Flavobacteriaceae;__Pibocella
70	Bacteria;__Bacteroidetes;__Flavobacteria;__Flavobacteriales;__Flavobacteriaceae;__Polaribacter
71	Bacteria;__Bacteroidetes;__Flavobacteria;__Flavobacteriales;__Flavobacteriaceae;__Tenacibaculum
72	Bacteria;__Bacteroidetes;__Flavobacteria;__Flavobacteriales;__Flavobacteriaceae;__Ulvibacter
73	Bacteria;__Bacteroidetes;__SB-1;__o;__f;__g
74	Bacteria;__Bacteroidetes;__Sphingobacteriia;__Sphingobacteriales;__Chitinophagaceae;__Hydrotalea
75	Bacteria;__Bacteroidetes;__Sphingobacteriia;__Sphingobacteriales;__Sphingobacteriaceae;__Sphingobacterium
76	Bacteria;__Bacteroidetes;__Sphingobacteriia;__Sphingobacteriales;__WCHB1-69;__g
77	Bacteria;__Bacteroidetes;__VC2.1_Bac22;__o;__f;__g
78	Bacteria;__Candidate_division_BRC1;__c;__o;__f;__g
79	Bacteria;__Candidate_division_OP3;__c;__o;__f;__g
80	Bacteria;__Candidate_division_SR1;__c;__o;__f;__g
81	Bacteria;__Chlamydiae;__Chlamydiae;__Chlamydiales;__Simkaniaceae;__Candidatus_Fritschea
82	Bacteria;__Chlamydiae;__Chlamydiae;__Chlamydiales;__Simkaniaceae;__g
83	Bacteria;__Chloroflexi;Other;Other;Other;Other
84	Bacteria;__Chloroflexi;__SAR202_clade;__o;__f;__g
85	Bacteria;__Cyanobacteria;__ML635J-21;__o;__f;__g
86	Bacteria;__Cyanobacteria;__SHA-109;__o;__f;__g
87	Bacteria;__Cyanobacteria;__SM2F09;__o;__f;__g
88	Bacteria;__Deferribacteres;__Deferribacteres;__Deferribacterales;__PAUC34f;__g
89	Bacteria;__Deferribacteres;__Deferribacteres;__Deferribacterales;__SAR406_clade(Marine_group_A);__g
90	Bacteria;__Fibrobacteres;__Fibrobacteria;__P.palm_C70;__f;__g
91	Bacteria;__Firmicutes;__Bacilli;__Bacillales;__Bacillaceae;__Bacillus
92	Bacteria;__Firmicutes;__Bacilli;__Bacillales;__Bacillaceae;__Marinococcus
93	Bacteria;__Firmicutes;__Bacilli;__Bacillales;__Family_XII_Incertae_Sedis;__Exiguobacterium
94	Bacteria;__Firmicutes;__Bacilli;__Lactobacillales;__Lactobacillaceae;__Lactobacillus
95	Bacteria;__Firmicutes;__Clostridia;__Clostridiales;__Clostridiaceae;__Clostridium
96	Bacteria;__Firmicutes;__Clostridia;__Clostridiales;__Family_XIII_Incertae_Sedis;Other
97	Bacteria;__Firmicutes;__Clostridia;__Clostridiales;__Family_XIII_Incertae_Sedis;__Incertae_Sedis
98	Bacteria;__Firmicutes;__Clostridia;__Clostridiales;__Family_XII_Incertae_Sedis;__Fusibacter
99	Bacteria;__Firmicutes;__Clostridia;__Clostridiales;__JTB215;__g
100	Bacteria;__Firmicutes;__Clostridia;__Clostridiales;__Lachnospiraceae;__Incertae_Sedis
101	Bacteria;__Firmicutes;__Clostridia;__Clostridiales;__Lachnospiraceae;__Pseudobutyrivibrio
102	Bacteria;__Firmicutes;__Clostridia;__Clostridiales;__Lachnospiraceae;__g
103	Bacteria;__Firmicutes;__Clostridia;__Clostridiales;__Peptococcaceae;__Peptococcus

104	Bacteria;__Firmicutes;__Clostridia;__Clostridiales;__Peptococcaceae;__g
105	Bacteria;__Firmicutes;__Clostridia;__Clostridiales;__Peptostreptococcaceae;__Incertae_Sedis
106	Bacteria;__Firmicutes;__Clostridia;__Clostridiales;__Ruminococcaceae;__Faecalibacterium
107	Bacteria;__Firmicutes;__Clostridia;__Clostridiales;__Ruminococcaceae;__g
108	Bacteria;__Firmicutes;__Clostridia;__Clostridiales;__Veillonellaceae;__Phascolarctobacterium
109	Bacteria;__Firmicutes;__Erysipelotrichi;__Erysipelotrichales;__Erysipelotrichaceae;__Turicibacter
110	Bacteria;__Fusobacteria;__Fusobacteria;__Fusobacteriales;__Fusobacteriaceae;__Propionigenium
111	Bacteria;__Fusobacteria;__Fusobacteria;__Fusobacteriales;__Fusobacteriaceae;__Psychrilyobacter
112	Bacteria;__Fusobacteria;__Fusobacteria;__Fusobacteriales;__Leptotrichiaceae;__g
113	Bacteria;__Gemmatimonadetes;__Gemmatimonadetes;__BD2-11_terrestrial_group;__f;__g
114	Bacteria;__Gemmatimonadetes;__Gemmatimonadetes;__Gemmatimonadales;__Gemmatimonadaceae;__Gemmatimonas
115	Bacteria;__Gemmatimonadetes;__Gemmatimonadetes;__PAUC43f_marine_benthic_group;__f;__g
116	Bacteria;__Lentisphaerae;__Lentisphaeria;__Lentisphaerales;__Lentisphaeraceae;__Lentisphaera
117	Bacteria;__Lentisphaerae;__Lentisphaeria;__MSBL3;__f;__g
118	Bacteria;__Lentisphaerae;__Lentisphaeria;__R76-B128;__f;__g
119	Bacteria;__Lentisphaerae;__Lentisphaeria;__Victivallales;__Victivallaceae;__g
120	Bacteria;__Lentisphaerae;__Lentisphaeria;__WCHB1-41;__f;__g
121	Bacteria;__Lentisphaerae;__Lentisphaeria;__c5LKS8;__f;__g
122	Bacteria;__Nitrospirae;__Nitrospira;__Nitrospirales;__Nitrospiraceae;__Leptospirillum
123	Bacteria;__Planctomycetes;__028H05-P-BN-P5;__o;__f;__g
124	Bacteria;__Planctomycetes;__BD7-11;__o;__f;__g
125	Bacteria;__Planctomycetes;__Phycisphaerae;__CCM11a;__f;__g
126	Bacteria;__Planctomycetes;__Phycisphaerae;__MSBL9;__f;__g
127	Bacteria;__Planctomycetes;__Phycisphaerae;__Phycisphaerales;__Phycisphaeraceae;__CL500-3
128	Bacteria;__Planctomycetes;__Phycisphaerae;__Phycisphaerales;__Phycisphaeraceae;__FS140-16B-02_marine_group
129	Bacteria;__Planctomycetes;__Phycisphaerae;__Phycisphaerales;__Phycisphaeraceae;__JL-ETNP-F27
130	Bacteria;__Planctomycetes;__Phycisphaerae;__Phycisphaerales;__Phycisphaeraceae;__Urania-1B-19_marine_sediment_group
131	Bacteria;__Planctomycetes;__Phycisphaerae;__Pla1_lineage;__f;__g
132	Bacteria;__Planctomycetes;__Phycisphaerae;__SHA-43;__f;__g
133	Bacteria;__Planctomycetes;__Pla3_lineage;__o;__f;__g
134	Bacteria;__Planctomycetes;__Pla4_lineage;__o;__f;__g
135	Bacteria;__Planctomycetes;__Planctomycetacia;__Planctomycetales;__Planctomycetaceae;__Blastopirellula
136	Bacteria;__Planctomycetes;__SGST604;__o;__f;__g
137	Bacteria;__Planctomycetes;__vadinHA49;__o;__f;__g
138	Bacteria;__Proteobacteria;__AEGEAN-245;__o;__f;__g
139	Bacteria;__Proteobacteria;__Alphaproteobacteria;Other;Other;Other
140	Bacteria;__Proteobacteria;__Alphaproteobacteria;__Caulobacterales;__Caulobacteraceae;__Amorphus
141	Bacteria;__Proteobacteria;__Alphaproteobacteria;__Caulobacterales;__Caulobacteraceae;__g
142	Bacteria;__Proteobacteria;__Alphaproteobacteria;__Caulobacterales;__Hyphomonadaceae;Other
143	Bacteria;__Proteobacteria;__Alphaproteobacteria;__Caulobacterales;__Hyphomonadaceae;__Hyphomonas
144	Bacteria;__Proteobacteria;__Alphaproteobacteria;__Caulobacterales;__Hyphomonadaceae;__Robiginitomaculum
145	Bacteria;__Proteobacteria;__Alphaproteobacteria;__Caulobacterales;__Hyphomonadaceae;__g
146	Bacteria;__Proteobacteria;__Alphaproteobacteria;__Kordiimonadales;__Kordiimonadaceae;__Kordiimonas
147	Bacteria;__Proteobacteria;__Alphaproteobacteria;__MNG3;__f;__g
148	Bacteria;__Proteobacteria;__Alphaproteobacteria;__OCS116_clade;__f;__g
149	Bacteria;__Proteobacteria;__Alphaproteobacteria;__Rhizobiales;__Bradyrhizobiaceae;__Rhodopseudomonas
150	Bacteria;__Proteobacteria;__Alphaproteobacteria;__Rhizobiales;__Hyphomicrobiaceae;__Maritalea
151	Bacteria;__Proteobacteria;__Alphaproteobacteria;__Rhizobiales;__KF-JG30-B3;__g
152	Bacteria;__Proteobacteria;__Alphaproteobacteria;__Rhizobiales;__Phyllobacteriaceae;__Cohaesibacter
153	Bacteria;__Proteobacteria;__Alphaproteobacteria;__Rhizobiales;__Phyllobacteriaceae;__Hoeflea
154	Bacteria;__Proteobacteria;__Alphaproteobacteria;__Rhizobiales;__Phyllobacteriaceae;__Nitratireductor
155	Bacteria;__Proteobacteria;__Alphaproteobacteria;__Rhizobiales;__Rhodobiaceae;__Rhodobium
156	Bacteria;__Proteobacteria;__Alphaproteobacteria;__Rhodobacterales;__Rhodobacteraceae;__Litoreibacter
157	Bacteria;__Proteobacteria;__Alphaproteobacteria;__Rhodobacterales;__Rhodobacteraceae;__Pseudovibrio
158	Bacteria;__Proteobacteria;__Alphaproteobacteria;__Rhodobacterales;__Rhodobacteraceae;__Roseobacter_clade_NAC11-7_lineage
159	Bacteria;__Proteobacteria;__Alphaproteobacteria;__Rhodobacterales;__Rhodobacteraceae;__Roseovarius
160	Bacteria;__Proteobacteria;__Alphaproteobacteria;__Rhodospirillales;__Acetobacteraceae;Other
161	Bacteria;__Proteobacteria;__Alphaproteobacteria;__Rhodospirillales;__Rhodospirillaceae;__AEGEAN-169_marine_group
162	Bacteria;__Proteobacteria;__Alphaproteobacteria;__Rhodospirillales;__Rhodospirillaceae;__Defluviicoccus
163	Bacteria;__Proteobacteria;__Alphaproteobacteria;__Rhodospirillales;__Rhodospirillaceae;__Nisaea
164	Bacteria;__Proteobacteria;__Alphaproteobacteria;__Rhodospirillales;__Rhodospirillaceae;__Pelagibius
165	Bacteria;__Proteobacteria;__Alphaproteobacteria;__Rhodospirillales;__Rhodospirillaceae;__Rhodospirillum
166	Bacteria;__Proteobacteria;__Alphaproteobacteria;__Rhodospirillales;__Rhodospirillaceae;__Thalassobaculum
167	Bacteria;__Proteobacteria;__Alphaproteobacteria;__Rhodospirillales;__Rhodospirillaceae;__Thalassospira
168	Bacteria;__Proteobacteria;__Alphaproteobacteria;__Rhodospirillales;__Rhodospirillaceae;__Tistrella
169	Bacteria;__Proteobacteria;__Alphaproteobacteria;__Rickettsiales;Other;Other
170	Bacteria;__Proteobacteria;__Alphaproteobacteria;__Rickettsiales;__Candidatus_Captivus;__g
171	Bacteria;__Proteobacteria;__Alphaproteobacteria;__Rickettsiales;__Candidatus_Hepatincola;__g
172	Bacteria;__Proteobacteria;__Alphaproteobacteria;__Rickettsiales;__Family_Incertae_Sedis;__Caedibacter
173	Bacteria;__Proteobacteria;__Alphaproteobacteria;__Rickettsiales;__Ho(lab);__g
174	Bacteria;__Proteobacteria;__Alphaproteobacteria;__Rickettsiales;__Holosporaceae;__Holospora
175	Bacteria;__Proteobacteria;__Alphaproteobacteria;__Rickettsiales;__LWSR-14;__g
176	Bacteria;__Proteobacteria;__Alphaproteobacteria;__Rickettsiales;__RB446;__g
177	Bacteria;__Proteobacteria;__Alphaproteobacteria;__Rickettsiales;__Rickettsiaceae;__Candidatus_Cryptoprodotis
178	Bacteria;__Proteobacteria;__Alphaproteobacteria;__Rickettsiales;__Rickettsiaceae;__Rickettsia
179	Bacteria;__Proteobacteria;__Alphaproteobacteria;__Rickettsiales;__S25-593;__g
180	Bacteria;__Proteobacteria;__Alphaproteobacteria;__Rickettsiales;__SAR116_clade;__g
181	Bacteria;__Proteobacteria;__Alphaproteobacteria;__Rickettsiales;__SM2D12;__g
182	Bacteria;__Proteobacteria;__Alphaproteobacteria;__Rickettsiales;__TK34;__g
183	Bacteria;__Proteobacteria;__Alphaproteobacteria;__S26-47;__f;__g
184	Bacteria;__Proteobacteria;__Alphaproteobacteria;__SAR11_clade;__Deep_1;__g
185	Bacteria;__Proteobacteria;__Alphaproteobacteria;__SAR11_clade;__Surface_1;__g
186	Bacteria;__Proteobacteria;__Alphaproteobacteria;__SAR11_clade;__Surface_4;__g
187	Bacteria;__Proteobacteria;__Alphaproteobacteria;__SB1-18;__f;__g
188	Bacteria;__Proteobacteria;__Alphaproteobacteria;__Sphingomonadales;__Erythrobacteraceae;__Altererythrobacter
189	Bacteria;__Proteobacteria;__Alphaproteobacteria;__Sphingomonadales;__Erythrobacteraceae;__g
190	Bacteria;__Proteobacteria;__Alphaproteobacteria;__Sphingomonadales;__Family_Incertae_Sedis;__Kiloniella
191	Bacteria;__Proteobacteria;__Alphaproteobacteria;__Sphingomonadales;__GOBB3-C201;__g
192	Bacteria;__Proteobacteria;__Alphaproteobacteria;__Sphingomonadales;__Sphingomonadaceae;Other
193	Bacteria;__Proteobacteria;__Alphaproteobacteria;__Sphingomonadales;__Sphingomonadaceae;__Novosphingobium
194	Bacteria;__Proteobacteria;__Alphaproteobacteria;__Sphingomonadales;__Sphingomonadaceae;__Sphingomonas
195	Bacteria;__Proteobacteria;__Alphaproteobacteria;__Sphingomonadales;__Sphingomonadaceae;__Sphingopyxis
196	Bacteria;__Proteobacteria;__Betaproteobacteria;__Burkholderiales;__Alcaligenaceae;__Sutterella
197	Bacteria;__Proteobacteria;__Betaproteobacteria;__Hydrogenophilales;__Hydrogenophilaceae;__Thiobacillus
198	Bacteria;__Proteobacteria;__Betaproteobacteria;__Methylophilales;__Methylophilaceae;__OM43_clade
199	Bacteria;__Proteobacteria;__Betaproteobacteria;__Neisseriales;__Neisseriaceae;__g
200	Bacteria;__Proteobacteria;__Class_Incertae_Sedis;__Order_Incertae_Sedis;__Family_Incertae_Sedis;__Mariprofundus
201	Bacteria;__Proteobacteria;__Deltaproteobacteria;Other;Other;Other
202	Bacteria;__Proteobacteria;__Deltaproteobacteria;__Bdellovibrionales;__Bacteriovoracaceae;Other
203	Bacteria;__Proteobacteria;__Deltaproteobacteria;__Bdellovibrionales;__Bacteriovoracaceae;__g
204	Bacteria;__Proteobacteria;__Deltaproteobacteria;__Bdellovibrionales;__Bdellovibrionaceae;__Bdellovibrio
205	Bacteria;__Proteobacteria;__Deltaproteobacteria;__Bdellovibrionales;__Bdellovibrionaceae;__OM27_clade
206	Bacteria;__Proteobacteria;__Deltaproteobacteria;__Desulfarculales;__Desulfarculaceae;__g

207	Bacteria;__Proteobacteria;__Deltaproteobacteria;__Desulfobacterales;__Desulfobacteraceae;__Desulfofrigus
208	Bacteria;__Proteobacteria;__Deltaproteobacteria;__Desulfobacterales;__Desulfobacteraceae;__SEEP-SRB1
209	Bacteria;__Proteobacteria;__Deltaproteobacteria;__Desulfobacterales;__Desulfobulbaceae;__Desulfobulbus
210	Bacteria;__Proteobacteria;__Deltaproteobacteria;__Desulfobacterales;__Desulfobulbaceae;__Desulfocapsa
211	Bacteria;__Proteobacteria;__Deltaproteobacteria;__Desulfobacterales;__Desulfobulbaceae;__Desulforhopalus
212	Bacteria;__Proteobacteria;__Deltaproteobacteria;__Desulfobacterales;__Desulfobulbaceae;__g
213	Bacteria;__Proteobacteria;__Deltaproteobacteria;__Desulfobacterales;__Nitrospinaceae;__Nitrospina
214	Bacteria;__Proteobacteria;__Deltaproteobacteria;__Desulfobacterales;__Nitrospinaceae;__g
215	Bacteria;__Proteobacteria;__Deltaproteobacteria;__Desulfovibrionales;__Desulfovibrionaceae;__Desulfovibrio
216	Bacteria;__Proteobacteria;__Deltaproteobacteria;__Desulfuromonadales;__Desulfuromonadaceae;__Desulfuromusa
217	Bacteria;__Proteobacteria;__Deltaproteobacteria;__Desulfuromonadales;__Sva1033;__g
218	Bacteria;__Proteobacteria;__Deltaproteobacteria;__Myxococcales;__Cystobacterineae;__g
219	Bacteria;__Proteobacteria;__Deltaproteobacteria;__Myxococcales;__Haliangiaceae;__Haliangium
220	Bacteria;__Proteobacteria;__Deltaproteobacteria;__Myxococcales;__Nannocystaceae;Other
221	Bacteria;__Proteobacteria;__Deltaproteobacteria;__Myxococcales;__Nannocystaceae;__Enhygromyxa
222	Bacteria;__Proteobacteria;__Deltaproteobacteria;__Myxococcales;__Nannocystaceae;__g
223	Bacteria;__Proteobacteria;__Deltaproteobacteria;__Myxococcales;__Nannocystineae;__g
224	Bacteria;__Proteobacteria;__Deltaproteobacteria;__Myxococcales;__Sorangiineae;__Sandaracinaceae
225	Bacteria;__Proteobacteria;__Deltaproteobacteria;__SAR324_clade(Marine_group_B);__f;__g
226	Bacteria;__Proteobacteria;__Deltaproteobacteria;__Sva0485;__f;__g
227	Bacteria;__Proteobacteria;__Epsilonproteobacteria;__Campylobacterales;__Campylobacteraceae;__Arcobacter
228	Bacteria;__Proteobacteria;__Epsilonproteobacteria;__Campylobacterales;__Campylobacteraceae;__Sulfurospirillum
229	Bacteria;__Proteobacteria;__Epsilonproteobacteria;__Campylobacterales;__Helicobacteraceae;__Sulfurimonas
230	Bacteria;__Proteobacteria;__Gammaproteobacteria;Other;Other;Other
231	Bacteria;__Proteobacteria;__Gammaproteobacteria;__34P16;__f;__g
232	Bacteria;__Proteobacteria;__Gammaproteobacteria;__Alteromonadales;Other;Other
233	Bacteria;__Proteobacteria;__Gammaproteobacteria;__Alteromonadales;__Alteromonadaceae;Other
234	Bacteria;__Proteobacteria;__Gammaproteobacteria;__Alteromonadales;__Alteromonadaceae;__Agarivorans
235	Bacteria;__Proteobacteria;__Gammaproteobacteria;__Alteromonadales;__Alteromonadaceae;__C1-B045
236	Bacteria;__Proteobacteria;__Gammaproteobacteria;__Alteromonadales;__Alteromonadaceae;__Dasania
237	Bacteria;__Proteobacteria;__Gammaproteobacteria;__Alteromonadales;__Alteromonadaceae;__OM60(NOR5)_clade
238	Bacteria;__Proteobacteria;__Gammaproteobacteria;__Alteromonadales;__Alteromonadaceae;__Simiduia
239	Bacteria;__Proteobacteria;__Gammaproteobacteria;__Alteromonadales;__Alteromonadaceae;__g
240	Bacteria;__Proteobacteria;__Gammaproteobacteria;__Alteromonadales;__Colwelliaceae;__Thalassomonas
241	Bacteria;__Proteobacteria;__Gammaproteobacteria;__Alteromonadales;__Moritellaceae;__Moritella
242	Bacteria;__Proteobacteria;__Gammaproteobacteria;__Alteromonadales;__Pseudoalteromonadaceae;__Pseudoalteromonas
243	Bacteria;__Proteobacteria;__Gammaproteobacteria;__Alteromonadales;__Psychromonadaceae;__Psychromonas
244	Bacteria;__Proteobacteria;__Gammaproteobacteria;__Alteromonadales;__Shewanellaceae;__Shewanella
245	Bacteria;__Proteobacteria;__Gammaproteobacteria;__Chromatiales;Other;Other
246	Bacteria;__Proteobacteria;__Gammaproteobacteria;__Chromatiales;__Chromatiaceae;__Nitrosococcus
247	Bacteria;__Proteobacteria;__Gammaproteobacteria;__Chromatiales;__Ectothiorhodospiraceae;__Acidiferrobacter
248	Bacteria;__Proteobacteria;__Gammaproteobacteria;__Chromatiales;__Ectothiorhodospiraceae;__Thioalkalispira
249	Bacteria;__Proteobacteria;__Gammaproteobacteria;__Chromatiales;__Halothiobacillaceae;__Halothiobacillus
250	Bacteria;__Proteobacteria;__Gammaproteobacteria;__E01-9C-26_marine_group;__f;__g
251	Bacteria;__Proteobacteria;__Gammaproteobacteria;__EC3;__f;__g
252	Bacteria;__Proteobacteria;__Gammaproteobacteria;__KI89A_clade;__f;__g
253	Bacteria;__Proteobacteria;__Gammaproteobacteria;__Legionellales;__Coxiellaceae;__Coxiella
254	Bacteria;__Proteobacteria;__Gammaproteobacteria;__Legionellales;__Coxiellaceae;__Rickettsiella
255	Bacteria;__Proteobacteria;__Gammaproteobacteria;__Legionellales;__Coxiellaceae;__g
256	Bacteria;__Proteobacteria;__Gammaproteobacteria;__Legionellales;__Legionellaceae;__g
257	Bacteria;__Proteobacteria;__Gammaproteobacteria;__Methylococcales;__Crenotrichaceae;__Crenothrix
258	Bacteria;__Proteobacteria;__Gammaproteobacteria;__Methylococcales;__Methylococcaceae;__Methylosoma
259	Bacteria;__Proteobacteria;__Gammaproteobacteria;__Oceanospirillales;Other;Other
260	Bacteria;__Proteobacteria;__Gammaproteobacteria;__Oceanospirillales;__CrystalBog021C3;__g
261	Bacteria;__Proteobacteria;__Gammaproteobacteria;__Oceanospirillales;__Halomonadaceae;__Salinicola
262	Bacteria;__Proteobacteria;__Gammaproteobacteria;__Oceanospirillales;__OM182_clade;__g
263	Bacteria;__Proteobacteria;__Gammaproteobacteria;__Oceanospirillales;__Oceanospirillaceae;__Marinospirillum
264	Bacteria;__Proteobacteria;__Gammaproteobacteria;__Oceanospirillales;__SAR86_clade;__g
265	Bacteria;__Proteobacteria;__Gammaproteobacteria;__Oceanospirillales;__SS1-B-06-26;__g
266	Bacteria;__Proteobacteria;__Gammaproteobacteria;__Oceanospirillales;__Saccharospirillaceae;__Saccharospirillum
267	Bacteria;__Proteobacteria;__Gammaproteobacteria;__Order_Incertae_Sedis;__Family_Incertae_Sedis;__Arenicella
268	Bacteria;__Proteobacteria;__Gammaproteobacteria;__Order_Incertae_Sedis;__Family_Incertae_Sedis;__Sedimenticola
269	Bacteria;__Proteobacteria;__Gammaproteobacteria;__Pseudomonadales;__Moraxellaceae;__Acinetobacter
270	Bacteria;__Proteobacteria;__Gammaproteobacteria;__Pseudomonadales;__Moraxellaceae;__Enhydrobacter
271	Bacteria;__Proteobacteria;__Gammaproteobacteria;__Salinisphaerales;__Salinisphaeraceae;__Salinisphaera
272	Bacteria;__Proteobacteria;__Gammaproteobacteria;__Salinisphaerales;__Salinisphaeraceae;__ZD0417_marine_group
273	Bacteria;__Proteobacteria;__Gammaproteobacteria;__Thiotrichales;Other;Other
274	Bacteria;__Proteobacteria;__Gammaproteobacteria;__Thiotrichales;__1G93;__g
275	Bacteria;__Proteobacteria;__Gammaproteobacteria;__Thiotrichales;__CHAB-XI-27;__g
276	Bacteria;__Proteobacteria;__Gammaproteobacteria;__Thiotrichales;__EV818SWSAP88;__g
277	Bacteria;__Proteobacteria;__Gammaproteobacteria;__Thiotrichales;__Family_Incertae_Sedis;__Caedibacter
278	Bacteria;__Proteobacteria;__Gammaproteobacteria;__Thiotrichales;__Piscirickettsiaceae;__Cycloclasticus
279	Bacteria;__Proteobacteria;__Gammaproteobacteria;__Thiotrichales;__Piscirickettsiaceae;__Methylophaga
280	Bacteria;__Proteobacteria;__Gammaproteobacteria;__Thiotrichales;__Piscirickettsiaceae;__Piscirickettsia
281	Bacteria;__Proteobacteria;__Gammaproteobacteria;__Thiotrichales;__Piscirickettsiaceae;__g
282	Bacteria;__Proteobacteria;__Gammaproteobacteria;__Thiotrichales;__Thiotrichaceae;__Candidatus_Thiopilula
283	Bacteria;__Proteobacteria;__Gammaproteobacteria;__Vibrionales;__Vibrionaceae;__Aliivibrio
284	Bacteria;__Proteobacteria;__Gammaproteobacteria;__Vibrionales;__Vibrionaceae;__g
285	Bacteria;__Proteobacteria;__Gammaproteobacteria;__Xanthomonadales;__Xanthomonadaceae;__Arenimonas
286	Bacteria;__Proteobacteria;__Gammaproteobacteria;__Xanthomonadales;__Xanthomonadaceae;__Luteibacter
287	Bacteria;__Proteobacteria;__Gammaproteobacteria;__Xanthomonadales;__Xanthomonadaceae;__g
288	Bacteria;__Proteobacteria;__JTB23;__o;__f;__g
289	Bacteria;__Proteobacteria;__Milano-WF1B-44;__o;__f;__g
290	Bacteria;__Proteobacteria;__Skagenf62;__o;__f;__g
291	Bacteria;__Proteobacteria;__TA18;__o;__f;__g
292	Bacteria;__RF3;__c;__o;__f;__g
293	Bacteria;__Spirochaetes;__Spirochaetes;__LK-44f;__f;__g
294	Bacteria;__Spirochaetes;__Spirochaetes;__MSBL8;__f;__g
295	Bacteria;__Spirochaetes;__Spirochaetes;__Spirochaetales;__Leptospiraceae;__Turneriella
296	Bacteria;__Spirochaetes;__Spirochaetes;__Spirochaetales;__Spirochaetaceae;__Spirochaeta
297	Bacteria;__Spirochaetes;__Spirochaetes;__Spirochaetales;__Spirochaetaceae;__Treponema
298	Bacteria;__Tenericutes;__Mollicutes;__Anaeroplasmatales;__Anaeroplasmataceae;__Anaeroplasma
299	Bacteria;__Tenericutes;__Mollicutes;__Mycoplasmatales;__Mycoplasmataceae;Other
300	Bacteria;__Verrucomicrobia;__Arctic97B-4_marine_group;__o;__f;__g
301	Bacteria;__Verrucomicrobia;__Opitutae;Other;Other;Other
302	Bacteria;__Verrucomicrobia;__Opitutae;__A714019;__f;__g
303	Bacteria;__Verrucomicrobia;__Opitutae;__MB11C04_marine_group;__f;__g
304	Bacteria;__Verrucomicrobia;__Opitutae;__Puniceicoccales;__Puniceicoccaceae;Other
305	Bacteria;__Verrucomicrobia;__Opitutae;__Puniceicoccales;__Puniceicoccaceae;__Cerasicoccus
306	Bacteria;__Verrucomicrobia;__Opitutae;__Puniceicoccales;__Puniceicoccaceae;__Pelagicoccus
307	Bacteria;__Verrucomicrobia;__Opitutae;__Puniceicoccales;__Puniceicoccaceae;__marine_group
308	Bacteria;__Verrucomicrobia;__Spartobacteria;__Chthoniobacterales;__FukuN18_freshwater_group;__g
309	Bacteria;__Verrucomicrobia;__Spartobacteria;__Chthoniobacterales;__Xiphinematobacteraceae;__Candidatus_Xiphinematobacter
310	Bacteria;__Verrucomicrobia;__Verrucomicrobiae;__Verrucomicrobiales;__Verrucomicrobiaceae;__Roseibacillus
